# Targeting Class I_A_ PI3K Isoforms Selectively Impairs Cell Growth, Survival, and Migration in Glioblastoma

**DOI:** 10.1371/journal.pone.0094132

**Published:** 2014-04-09

**Authors:** Katrin Höland, Danielle Boller, Christian Hagel, Silvia Dolski, András Treszl, Olivier E. Pardo, Paulina Ćwiek, Fabiana Salm, Zaira Leni, Peter R. Shepherd, Beata Styp-Rekowska, Valentin Djonov, André O. von Bueren, Karl Frei, Alexandre Arcaro

**Affiliations:** 1 Department of Clinical Research, University of Bern, Bern, Switzerland; 2 Division of Clinical Chemistry and Biochemistry, University Children’s Hospital Zurich, Zurich, Switzerland; 3 Institute of Neuropathology, University Medical Center Hamburg-Eppendorf, Hamburg, Germany; 4 Department of Neurosurgery, University Hospital Zurich, Zurich, Switzerland; 5 Department of Medical Biometry and Epidemiology, Center for Experimental Medicine, University Medical Center Hamburg-Eppendorf, Hamburg, Germany; 6 Department of Surgery and Cancer, Imperial College London, London, United Kingdom; 7 Department of Molecular Medicine and Pathology, University of Auckland, Auckland, New Zealand; 8 Institute of Anatomy, University of Bern, Bern, Switzerland; 9 Division of Pediatric Hematology and Oncology, Department of Pediatrics and Adolescent Medicine, University Medical Center Goettingen, Goettingen, Germany; University of Bari Medical School, Italy

## Abstract

The phosphoinositide 3-kinase (PI3K)/Akt/mammalian target of rapamycin (mTOR) pathway is frequently activated in human cancer and plays a crucial role in glioblastoma biology. We were interested in gaining further insight into the potential of targeting PI3K isoforms as a novel anti-tumor approach in glioblastoma. Consistent expression of the PI3K catalytic isoform PI3K p110α was detected in a panel of glioblastoma patient samples. In contrast, PI3K p110β expression was only rarely detected in glioblastoma patient samples. The expression of a module comprising the epidermal growth factor receptor (EGFR)/PI3K p110α/phosphorylated ribosomal S6 protein (p-S6) was correlated with shorter patient survival. Inhibition of PI3K p110α activity impaired the anchorage-dependent growth of glioblastoma cells and induced tumor regression *in vivo*. Inhibition of PI3K p110α or PI3K p110β also led to impaired anchorage-independent growth, a decreased migratory capacity of glioblastoma cells, and reduced the activation of the Akt/mTOR pathway. These effects were selective, because targeting of PI3K p110δ did not result in a comparable impairment of glioblastoma tumorigenic properties. Together, our data reveal that drugs targeting PI3K p110α can reduce growth in a subset of glioblastoma tumors characterized by the expression of EGFR/PI3K p110α/p-S6.

## Introduction

Gliomas are the most common tumors of the central nervous system, accounting for 80% of malignant brain tumors. Among these, glioblastoma (GBM) represents the largest and most malignant subgroup. GBMs are generally characterized by a highly heterogeneous and highly infiltrative phenotype. Standard treatment of care includes surgical resection, followed by radiation and/or chemotherapy [1]. Despite this aggressive treatment, almost all GBM eventually recur and the median survival of GBM patients is only around one year [2], [3]. Novel therapies are thus urgently needed.

Genetic alterations in GBM predominantly include amplifications or mutations in the gene encoding the epidermal growth factor receptor (*EGFR*). A rather characteristic mutation thereby leads to the truncated version EGFRvIII, present in about 20% of GBM [4], which possesses an in-frame deletion of exons 2 to 7 in the extracellular domain and whose expression has been shown to correlate with elevated downstream signaling activation [5], [6]. Signaling via EGFR has also been demonstrated to contribute to resistance of GBM to radiation and chemotherapy [7]. Further growth factors have been described to play important roles in GBM tumorigenesis. For instance, insulin-like growth factor I (IGF-I) seems to be important in GBM chemoresistance, whereas platelet-derived growth factor (PDGF) is thought to signal in an autocrine fashion in GBM cells, thereby contributing to proliferation and survival of GBM tumor cells [8], [9]. Due to the fact that several different growth factors and their receptors play an important role in GBM biology, the treatment of one receptor alone might not lead to satisfying results. On the contrary, targeting a signaling molecule downstream of these growth factors might represent a successful treatment strategy.

The family of phosphoinositide 3-kinases (PI3Ks) lies downstream of several growth factor receptors. PI3Ks play an essential role in signal transmission and they are crucial in controlling cell proliferation, survival, and motility [10]. These enzymes phosphorylate membrane phosphoinositides on the inner side of the cell membrane on the D3 position of the inositol ring. These 3-phosphorylated membrane phosphoinositides then act as second messengers which are important for the activation of the downstream signaling pathway, including phosphorylation and thereby activation of Akt [11]. Further accounting to the importance of PI3K signaling in cancer is the fact that the tumor suppressor gene phosphatase and tensin homologue deleted on chromosome 10 (*PTEN*) is frequently mutated in human cancers, including GBM (approximately 20–30%) [10], [12]–[15]. PTEN acts as an antagonist of PI3K by dephosphorylating phosphatidylinositol-3,4,5-trisphosphate and thus reducing downstream signaling activity [16], [17]. Additionally, *PIK3CA*, the gene encoding class I_A_ PI3K isoform p110α, has been shown to be mutated in GBM tumor samples. *PIK3CA* mutations were found in 0–27% of GBM tumor samples, depending on the study and the detection method used [14], [15], [18]–[21]. Furthermore, and also depending on the study and the method used, copy number increases of both *PIK3CA* and *PIK3CD* (encoding PI3K p110δ) have been reported (ranging from approximately 0% to 60%) [14], [19], [21]–[23]. Taken together, deregulation of the PI3K/Akt/mTOR pathway is frequently detected in GBM (in around 88%) [12] and has been found to contribute to a variety of cellular responses, thus rendering this pathway and especially the PI3Ks interesting candidates for targeted therapies.

In the present study, we have investigated the expression of class I_A_ PI3K isoforms in GBM tumor samples, cell lines, and *ex vivo* cultures. We have further investigated the involvement of the different isoforms in cell proliferation, survival, and migration. Our results show that individual class I_A_ PI3K isoforms have selected cellular functions in GBM cells. The PI3K p110α had a broad expression pattern in primary tumors and was associated with the phosphorylation status of the ribosomal S6 protein, which correlated with decreased patient survival. The PI3K isoform p110α also appears to be essential for cell growth under anchorage-independent conditions and *in vivo*. Targeting PI3K p110β impaired anchorage-independent growth and cell migration in GBM cells, while targeting PI3K p110δ was ineffective. Thus the PI3K p110α and p110β isoforms appear to differentially contribute to critical cell responses in GBM, which is important when considering the future use of isoform-specific PI3K isoforms in this devastating malignancy.

## Materials and Methods

### Glioma Tissue Microarray

The study cohort comprised of low and high grade glioma samples from 103 patients operated at the Department of Neurosurgery, University Hospital Zurich (Zurich, Switzerland). Written informed consent was obtained from all the patients before study entry. The procedures were conducted in accordance with the Declaration of Helsinki and approved by the Ethics Committee of the Canton Zurich. Paraffin blocks of these tumors were reviewed by a neuropathologist and classified according to the World Health Organization (WHO) of brain tumors [24]. Representative tumor areas were marked on hematoxylin/eosin-stained slides and two cores (0.6 mm diameter) were punched from the donor block and transferred into the tissue microarray (TMA) recipient block, as recently described [25]. Additionally, the TMA contained four normal brain samples (in duplicates) and four cell lines (MCF-7, LN18, HT29, LN229). Normal brain tissue specimens served as control and were derived from autopsy material archived at the Institute of Neuropathology, University Hospital Zurich (Zurich, Switzerland). According to the Ethics Committee of the Canton Zurich, no ethical approval was necessary, as the tissue samples were anonymized and used for antibody validation and quality control of antibody immunostaings. MCF-7 and HT-29 were purchased from the American Type Culture Collection (Manassas, VA, USA) and the glioma cell lines LN18 and LN229 were kindly provided by Dr. N. de Tribolet (Lausanne, Switzerland) [26].

For immunohistochemistry (IHC) analysis freshly cut 3 μm thick sections of the TMA block were mounted on SuperFrost slides (Menzel Gläser, Braunschweig, Germany). Primary antibodies were incubated overnight at 4°C. The LSAB+System-HRP kit (DAKO, Glostrup, Denmark) was used for primary antibodies against PI3K p110α (1∶200), p-Akt (S473) (1∶50), p-S6 (S235/236) (1∶200) (all Cell Signaling, Danvers, MA, USA), and PI3K p110β (1∶100; Abcam, Cambridge, United Kingdom) according to the manufacturer’s protocol. For antibodies against EGFR (1∶50), Akt1 (1∶400), and S6 (1∶100) (all Cell Signaling, Danvers, MA, USA) N-Histofine Simple Stain MAX PO anti-rabbit (Nichirei Biosciences, Tokyo, Japan) was used as a secondary antibody. PTEN primary antibody (Cell Signaling, #9559, 1∶100) was applied in an automated stainer (Ventana Medical Systems, Tucson, AZ, USA) according to a standard protocol (pretreatment protocol “CC1m”). Bound antibodies were detected by the peroxidase method using diaminobenzidine as chromogen (760-500, Ultraview DAB Detection Kit; Ventana Medical Systems, Tuscon, AZ, USA). Finally, the slides were counterstained with hematoxylin prior to dehydration and coverslipping.

IHC labeling of tumor cells in all samples was evaluated semi-quantitatively by an experienced neuropathologist (C.H.) according to the following criteria: no staining (0), slight staining intensity of up to 30% of cells (1), slight to medium staining intensity of 30–50% of cells (2), medium to strong staining intensity of at least 50% of cells (3). Immunostaining scoring was performed in an entirely blinded fashion.

Cases with clinical follow-up were evaluated with respect to association of clinical factors and immunostaining scores to overall survival (OS). OS was defined as date of diagnosis to death of any cause or to the date of the last visit. Univariable analyses were performed by the Kaplan-Meier method, and the log-rank test was used for comparisons of survival in different groups [27]. Cox-regression analysis was applied to analyze the correlation between immunostaining and OS, adjusted for age, gender, and WHO grade (for this analysis immunostaining scores were dichotomized as immunopositive versus immunonegative). Further, the number of positive immunostainings was summarized for both cascades (EGFR, PI3K p110α/β, Akt1/p-Akt (S473)) and (PTEN, Akt1/p-Akt (S473)), with negative values for positive PTEN staining since PTEN antagonizes the activation of the signaling pathway. This sum score was then compared between S6 and p-S6 (S235/236) positive and negative patients, using permutation tests and bootstrap means based on 10′000 repetitions. All statistical analyses were intended to be rather exploratory than confirmatory and nominal p-values are reported, without adjustment for multiple testing. p-values <0.05, two-tailed are considered statistically significant. Statistical analyses were performed using SAS (Version 9.2 for Windows; SAS Institute Inc., Cary, NC, USA) and PASW Statistics 18 for Windows (SPSS Inc., Chicago, IL, USA).

### Cell Lines and ex vivo Cultures

GBM cell lines were obtained from the American Type Culture Collection and from Professor M. E. Hegi (CHUV, Lausanne, Switzerland). *PIK3CA* mutated GBM cell lines SK-MG-17 (V344G) and SK-MG-26 (H1074Y) were kindly provided by Professor G. Ritter (Ludwig Institute for Cancer Research, New York Branch at MSKCC, New York, NY, USA) and have been previously described [21]. GBM *ex vivo* cultures were established at the Department of Neurosurgery of the University Hospital Zurich (Zurich, Switzerland) as previously described [28]. All procedures conducted during the establishment of the *ex vivo* cultures were in accordance with the Declaration of Helsinki and approved by the ethics committee of the Canton Zurich. Informed written consent was obtained from all patients. All GBM cell lines and *ex vivo* cultures were cultured in Dulbecco’s modified Eagle’s medium (Sigma-Aldrich, Buchs, Switzerland) supplemented with 10% (v/v) heat-inactivated fetal calf serum, L-glutamine (Sigma-Aldrich, Buchs, Switzerland), penicillin/streptomycin (GIBCO, Life Technologies Europe, Zug, Switzerland), and 10 μg/ml gentamycin (Sigma-Aldrich, Buchs, Switzerland).

### Western Blotting

Protein expression was analyzed by immunoblotting as described previously [29] using antibodies against EGFR, PI3K p110β, PI3K p110δ, Akt1/2/3, caspase 3, ICAD, ERK (all Santa Cruz Biotechnology, Inc., Santa Cruz, CA, USA), PI3K p110α, p-Akt (S473), p-Akt (T308), p-S6 (S235/236), p-S6 (S240/244), S6, PARP, p-ERK (all Cell Signaling, Danvers, MA, USA), and β-actin (Sigma-Aldrich, Buchs, Switzerland). Western blot analysis was usually performed in duplicates or triplicates and representative blots are depicted.

### Pharmacological Treatments and RNA Interference

Growth factors EGF, PDGF, and IGF-1 were obtained from Calbiochem (Merck (Schweiz) AG, Zug, Switzerland). YM024 [30], PIK75 [31], TGX221 [32], and IC87114 [33] were generously provided by Professor S. P. Jackson (The Australian Centre for Blood Diseases, Monash University, Melbourne, Australia). A66 was previously described [34]. BEZ235 [35] was obtained from ChemieTek (Indianapolis, IN, USA). For growth factor stimulation, cells were pretreated with 0, 1 μM YM024, or 1 μM TGX221 for 1 h. GBM cells were transfected with siRNA pools (siGENOME SMART pool siRNA reagents; Dharmacon, Lafayette, CO, USA) targeting PI3K p110α, PI3K p110β, PI3K p110δ, Akt1, Akt2, Akt3, S6K1, or Rac1 using DharmaFECT siRNA transfection reagents (Dharmacon, Lafayette, CO, USA) according to the manufacturer’s protocol. Dharmacon’s siGENOME Non-Targeting siRNA Pool #2 (SCR) and TOX transfection control (TOX) were used as negative, non-targeting and positive transfection control, respectively.

### Cell Proliferation and Anchorage-independent Growth

Cell proliferation was assessed using the CellTiter 96 AQ_ueous_ One Solution Cell Proliferation Assay (Promega, Dübendorf, Switzerland) according to the manufacturer’s protocol. Anchorage-independent growth was analyzed using soft agar assay. In short, cells were grown in 0.35% agar supported on a 0.5% agar layer in 6-well plates. Cells were overlaid with medium or the appropriate treatment weekly. After 28 days, colonies were stained with crystal violet and the number of colonies was counted. Cell proliferation and anchorage-independent growth assays were performed in quadruplets and triplicates, respectively. Individual experiments were repeated up to three times, as indicated in the corresponding figure legends.

### Flow Cytometry

Apoptotic cells were assessed by flow cytometry as described previously [36]. Adherent and floating cells were collected, stained with FITC-labeled Annexin V (Biotium, Hayward, CA, USA) and propidium iodide (PI), and analyzed on a BD LSR II flow cytometer using BD FACSDiva software (Version 6.1.3; Becton Dickinson AG, Allschwil, Switzerland) and FlowJo software (Version 5.4+; Tree Star, Inc., Ashland, OR, USA). Flow cytometry analysis measurements were performed in singlet with three repetitions of individual measurements.

### Cell Migration

For *in vitro* wound healing assays, a scratch was introduced into a confluent layer of cells using a 200 μl pipette tip. The medium was replaced with the appropriate treatment medium and plates were further incubated. Photos were taken under a Nikon Eclipse TS100 microscope (4 × magnification) supplied with a Nikon DXM1200 digital camera (Nikon ACT-1 2.70 software). Open image area was quantified using TScratch software [37] and wound closure was calculated by (open image area before treatment - open image area after treatment). Random migration experiments were performed as described [38]. Cell migration experiments were performed in quadruplets and individual experiments were repeated up to three times, as indicated in the corresponding figure legends.

### Chick Chorioallantoic Membrane Assay

Fertilized chicken eggs (*gallus gallus*) from a local hatchery were incubated in a humidified incubator at 37°C. On embryonic day 3 (ED3), a window was opened in the shell and covered again with tape to continue incubation. On ED7, a silicon ring was placed on the chorioallantoic membrane (CAM), and around 3 million T98G cells in 20 μl PBS were applied after gentle laceration of the membrane. On ED10, photos were taken under a Leica M205 FA microscope (10 × magnification) supplied with a Canon EOS 5D Mark II camera (Canon EOS Utility software) and tumors were treated with 20 μl of PI3K p110α-specific inhibitor PIK75 for four consecutive days. On ED14, post-treatment photos were acquired and the embryos were sacrificed. Tumor volume was estimated by 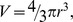 with 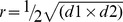 [39].

### Statistical Analysis of in vitro and in vivo Experiments

All statistical analyses of the *in vitro* and *in vivo* experiments were performed using Instat+ for Windows (Version 3.036; Statistical Services Centre, University of Reading, Reading, United Kingdom). Statistical significance between multiple groups was tested with ANOVA and p<0.05 was considered significant. Statistical significance between two groups was (subsequently) assessed by two-sided, two-sample Student’s *t*-test. For experiments where mean values were calculated over experiments with relative values two-sided, one-sample Student’s *t*-tests were performed. The individual statistical tests are indicated in the corresponding figure legends.

## Results

### Glioma TMA and Correlation Studies

**Figure 1:**
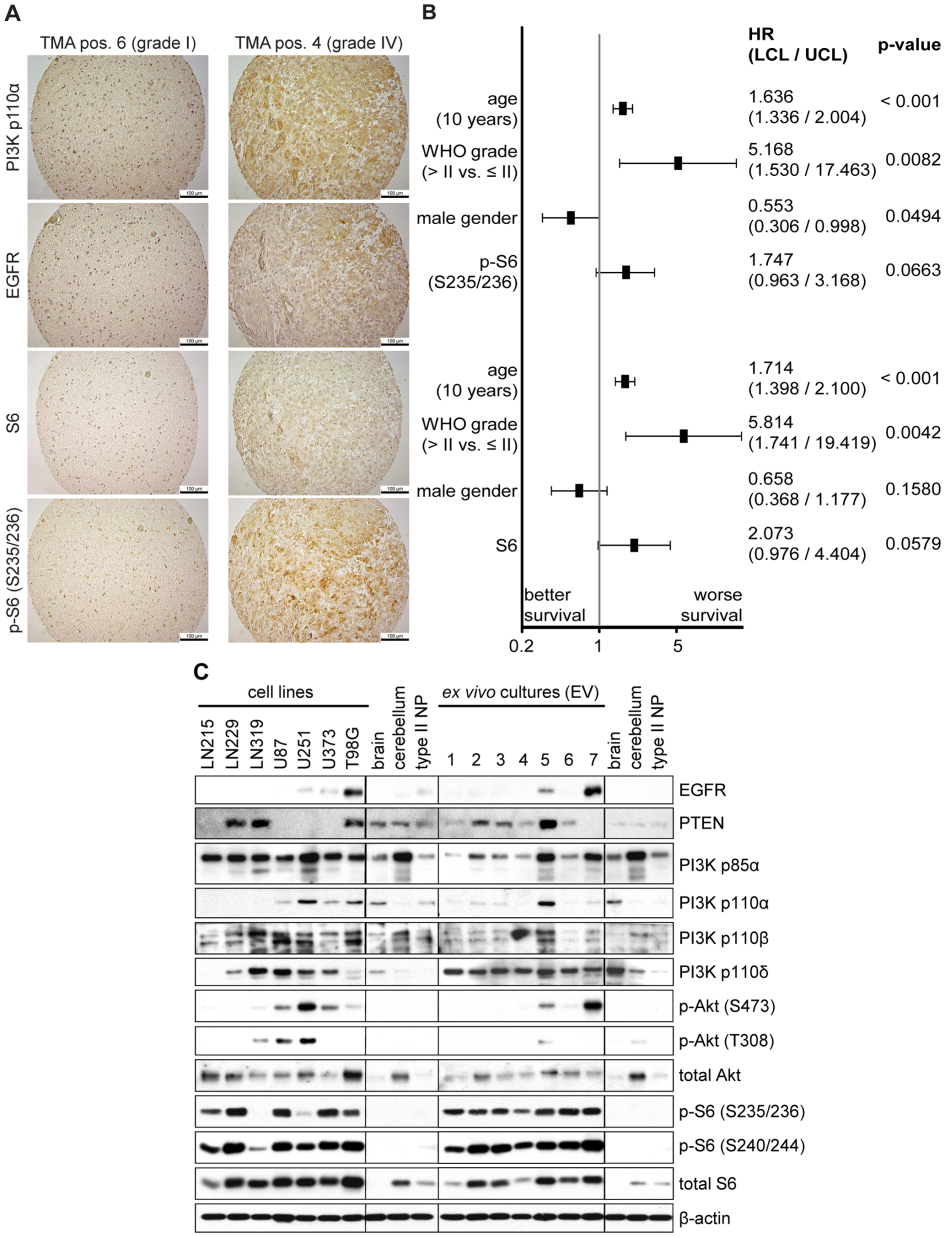
Expression analysis of proteins in the PI3K/Akt/mTOR signaling pathway in glioma and GBM samples. (A) IHC staining of glioma tissue microarray demonstrating increased expression of all antigens shown in GBM (WHO grade IV) compared to pilocytic astrocytoma (WHO grade I). Bars represent 100 μm. (B) Multivariable Cox-regression of IHC factors in 74 glioma patients by individually adding the immunostaining status to the clinical factors age, gender, and WHO grade. Results for p-S6 (S235/236) and S6 are shown in the upper and lower panel, respectively. HR: hazard ratio, LCL/UCL: lower/upper boundary of 95% confidence interval for HR. (C) Western blot analysis of protein levels of EGFR, PTEN, class I_A_ PI3K isoforms, and downstream signaling proteins Akt and S6 in human GBM cell lines and *ex vivo* cultures. Normal human brain and cerebellum tissue as well as non-transformed type II human pneumocytes were used as controls.

To investigate the expression of different components of the PI3K/Akt signaling pathway in human glioma tissue, we performed IHC analysis on a glioma TMA, containing samples of 103 patients, ranging from WHO grade I (pilocytic astrocytoma) to WHO grade IV (GBM) (Figure 1A and Table S1). Median follow-up time across all patients was 1.6 years (589 days). Complete disease information and IHC staining was available for 74 patients, while the other 29 patient were either lacking survival data or complete IHC staining. Demographics and characteristics of the 74 patients with complete information are summarized in Table S1.

Univariable analysis (Table S2) revealed that patients with high grade tumors (WHO grade>II) had a poorer 2-year OS when compared to patients with low grade tumors (WHO grade ≤ II; 32.2% versus 85.7%, p<0.001), which is consistent with previously described findings and the current state of knowledge [1].

Figure 1A shows examples of IHC staining with PI3K p110α, EGFR, S6, and p-S6 (S235/236) on two TMA positions, representing stainings of a WHO grade I pilocytic astrocytoma and a WHO grade IV glioblastoma and indicating increased expression of all antigens shown in case of GBM. The majority of glioma samples in the current study were immunopositive for PI3K p110α (73% of all glioma and 83% of GBM), while detection of PI3K p110β expression by IHC was infrequent (n = 1) and detection of PI3K p110δ was not possible due to the lack of suitable antibodies.

Immunopositive tumors for EGFR (80%) and p-S6 (S235/236) (42%) were frequently observed. The estimated 2-year OS for patients with p-S6 (S235/236) immunopositive tumors (22.6% versus 57.1%, p = 0.0043), with S6 immunopositive tumors (11.1% versus 46.9%, p = 0.0216), or with EGFR immunopositive tumors (OS 37.9% versus 60.0%, p = 0.0451) was less favorable when compared to patients with immunonegative tumors. Detection of p-Akt (S473) expression by IHC was infrequent (n = 1) in this particular cohort, while immunonegative tumors for PTEN were frequently observed (80%).

We next investigated the impact of immunostaining of molecular factors on OS adjusted for important clinical factors such as patients’ age, gender, and WHO grade. We added individually the immunostaining status to the clinical factors and applied multivariable Cox-regression analysis to estimate the effect of immunostaining. We found that there was a trend for S6 and p-S6 to be independently associated with worse OS when adjusted for important clinical factors. (Figure 1B;the effects fos p-S6 (S235/236) and S6 are shown in the upper and lower part, respectively).

To investigate which cascades (activation through EGFR and/or through PTEN) contribute to the downstream activation/phosphorylation of S6, we summarized the number of immunopositive staining of proteins in both cascades (EGFR, PI3K p110α/β, Akt1/p-Akt (S473)) and (PTEN, Akt1/p-Akt (S473)), using negative values for positive PTEN staining since PTEN antagonizes the activation of the PI3K/Akt/mTOR pathway. This analysis showed that patients with S6 positive tumors had higher scores in the first cascade (mean difference = 0.77, p = 0.0086) as well as in the second cascade (mean difference = 0.477, p = 0.0346). Contrary, patients with p-S6 (S235/236) positive tumors had higher score values in the first cascade (mean difference = 0.43, p = 0.0309) but not in the second cascade (mean difference = 0.2672, p = 0.0805), indicating that downstream signaling was altered in our patient cohort by EGFR/PI3K. The regulatory importance of PTEN was underlined when the scores of the first pathway were compared in patients without PTEN expression (PTEN IHC scoring = 0). In this subgroup of patients those with S6 positive tumors had lower scores in the first cascade (mean difference = 0.707, p = 0.0905); similarly p-S6 (S235/236) positive tumors had lower score values in the first cascade (mean difference = 0.48, p = 0.0660). Since the number of patients with PTEN expression (PTEN IHC scoring >0) was too small (n = 15), the cascade analysis could not be performed for this subgroup of patients.

Taken together, clinical factors (WHO-grade, gender, and age), and molecular factors (immunopositive tumors for S6 and p-S6 (S235/236)) are associated with OS. Our data provide evidence that the PI3K/mTOR/S6 signaling pathway can be activated through expression of EGFR/PI3K p110α rather than through reduced expression of PTEN in glioma.

### Expression of PI3K Isoforms in GBM Cell Lines and ex vivo Cultures

To gain an overview of the expression pattern of class I_A_ PI3K isoforms and the activation of the PI3K/Akt/mTOR signaling pathway in GBM cell lines and *ex vivo* cultures, we performed Western blot analysis on a set of seven GBM cell lines and seven *ex vivo* (EV) cultures (Figure 1C and Tables S3 and S4). Protein expression levels in GBM samples were compared to normal human brain and cerebellum tissue as well as to immortalized type II pneumocytes. While expression of the regulatory subunit PI3K p85α was comparable to levels observed in control lysates, differences in expression of the catalytic subunits PI3K p110α, PI3K p110β, and PI3K p110δ were found. PI3K p110α showed the most striking variation with some samples showing overexpression (U251, T98G, EV5), while expression of this isoform was nearly absent in certain cell lines (LN215, LN229, LN319) and *ex vivo* cultures (EV1-4, EV6-7). The expression of the PI3K isoform p110β was largely comparable to levels detected in control samples. Interestingly, PI3K p110δ was overexpressed in more than half of all GBM cell lines analyzed (LN319, U87, U251, U373), while the expression levels observed in *ex vivo* cultures were comparable to the controls. Basal activation of Akt was detected more frequently in cell lines (LN319, U87, U251, U373, T98G) than in *ex vivo* cultures (EV5, EV7) when compared to control samples. The most striking difference was found upon analysis of S6 protein activation. Strong basal phosphorylation of this protein was observed in all GBM cell lines and *ex vivo* cultures analyzed when compared to control lysates.

Taken together, this expression analysis revealed that individual catalytic class I_A_ PI3K isoforms are overexpressed in GBM cell lines and *ex vivo* cultures. Moreover, basal activation of the PI3K/Akt/mTOR pathway was detected in the samples analyzed. While phosphorylation of Akt was only observed in a subset of GBM cell lines and *ex vivo* cultures, activation of S6 protein was observed in all samples included in this study. These results confirm that the PI3K/Akt/mTOR signaling pathway is aberrantly activated in GBM.

### Inhibition of PI3K Isoform p110α Impairs GBM Cell Proliferation and Anchorage-Independent Growth

**Figure 2:**
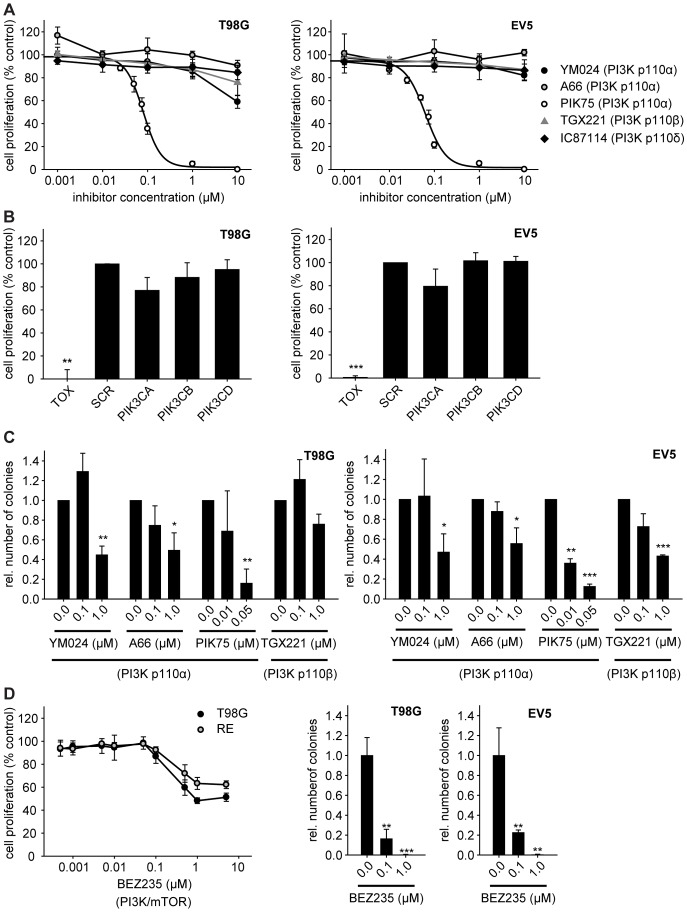
Inhibition of PI3K p110α impairs GBM cell proliferation and anchorage-independent growth. (A) Cell proliferation of GBM cells in the presence of class I_A_ PI3K isoform-specific inhibitors (72 h). (B) Cell proliferation of GBM cells transiently transfected with siRNA targeting class I_A_ PI3K isoforms p110α (PIK3CA), p110β (PIK3CB), or p110δ (PIK3CD) 48 h post transfection. TOX and SCR siRNAs were used as positive and negative, non-targeting controls, respectively. (C) Anchorage-independent growth (colony formation in soft agar) of GBM cells treated with PI3K p110α-specific inhibitors YM024, A66, PIK75, or PI3K p110β-specific inhibitor TGX221 (28 d). (D) Cell proliferation and anchorage-independent growth of GBM cells in the presence of the dual PI3K/mTOR inhibitor BEZ235 (72 h and 28 d, respectively). Curves and bars represent the means of three individual experiments ± standard deviation; single experiment for soft agar assay with BEZ235; *: p≤0.05, **: p≤0.01, ***: p≤0.001 compared to 0.0 μM inhibitor or SCR non-targeting siRNA control as determined by two-sided, one-sample Student’s *t*-tests.

Based on the importance of PI3K/Akt signaling and the deregulation of class I_A_ PI3K isoforms, especially PI3K p110α, we investigated the potential of pharmacological inhibition of theses isoforms in GBM cell proliferation. GBM cell lines and *ex vivo* cultures were treated with isoform-specific inhibitors YM024, A66, PIK75 (all PI3K p110α), TGX221 (PI3K p110β), or IC87114 (PI3K p110δ) for 72 h (Figure 2A and Figure S1A). Published IC_50_ values for PI3K p110 inhibition of these inhibitors are as follows: 300 nM for YM024 (PI3K p110α) [30], 32 nM for A66 (PI3K p110α) [34], 6–7.8 nM for PIK75 (PI3K p110α) [31], [34], 5–12 nM for TGX221 (PI3K p110β) [30]–[32], [34], and 41–500 nM for IC87114 (PI3K p110δ) [30], [31], [33], [34]. While inhibition of PI3K p110β or PI3K p110δ did not show any significant effect on cell proliferation of GBM cells, inhibition of PI3K p110α, especially when using inhibitor PIK75 (the inhibitor with the lowest *in vitro* IC_50_ value), led to a dose-dependent decrease in cell proliferation (Figure 2A and Figure S1A). To complement these studies, GBM cells were transiently transfected with siRNA targeting the three class I_A_ PI3K isoforms (*PIK3CA*, *PIK3CB*, or *PIK3CD*). The transfection with siRNAs led to a significant downregulation at the mRNA and protein level (Figure S2). In contrast to GBM cells with transiently downregulated PI3K p110β or PI3K p110δ, GBM cells transiently downregulated for PI3K p110α showed impaired cell proliferation (Figure 2B and Figure S1B). This confirms the results obtained with isoform-specific pharmacological inhibitors and shows that PI3K p110α contributes to GBM cell proliferation.

Since PI3K p110α-specific inhibitors have been shown to possess greater activity against tumor cell lines with a *PIK3CA* mutated background [40], we analyzed cell proliferation in the two *PIK3CA* mutated GBM cell lines SK-MG-17 (V344G) and SK-MG-26 (H1047Y) in the presence of either one of the three PI3K p110α-specific inhibitors YM024, PIK75, or A66 (Figure S3). The two cell lines possess different *PIK3CA* mutations: SK-MG-26 (H1047Y) possesses a rare mutation at a hot spot in the C-terminal portion of the kinase domain (H1047) which is known to lead to a gain of function, while SK-MG-17 (V344G) possesses a rare and not well characterized mutation in the C2 domain which is thought to be part of the membrane-binding region of PI3K p110α [21], [41]. When compared to the *PIK3CA* wild type GBM cell line T98G, SK-MG-26 (H1047Y) displayed a higher sensitivity towards the PI3K p110α-specific inhibitors, which was, however, not the case for SK-MG-17 (V344G).

Furthermore, anchorage-independent growth of GBM cells was analyzed by means of a soft agar assay in the presence of class I_A_ PI3K isoform-specific inhibitors. Treatment with three different PI3K p110α-specific inhibitors (YM024, A66, or PIK75) led to a concentration-dependent decrease in the ability of GBM cells to form colonies in soft agar (Figure 2C). Inhibition of PI3K p110β (TGX221) also decreased the number of colonies formed (Figure 2C), whereas the inhibition of PI3K p110δ (IC87114; Figure S4A) did not show significant effects on colony formation in soft agar. These observations indicate that the PI3K isoforms p110α and PI3K p110β play an important role in anchorage-independent growth of GBM cells, and thus potentially in their tumorigenic potential.

We further investigated cell proliferation and anchorage-independent growth of GBM cells in the presence of the dual PI3K/mTOR inhibitor BEZ235 (Figure 2D). A comparison of these results with the PI3K p110α-specific inhibitors showed that cell proliferation and colony formation in soft agar can be significantly impaired by only inhibiting the PI3K isoform p110α, instead of a combined inhibition of PI3K p110α and mTOR. Thus, concomitant inhibition of PI3K p110α and mTOR might not be necessary to impair growth in all GBMs.

### Apoptosis

**Figure 3:**
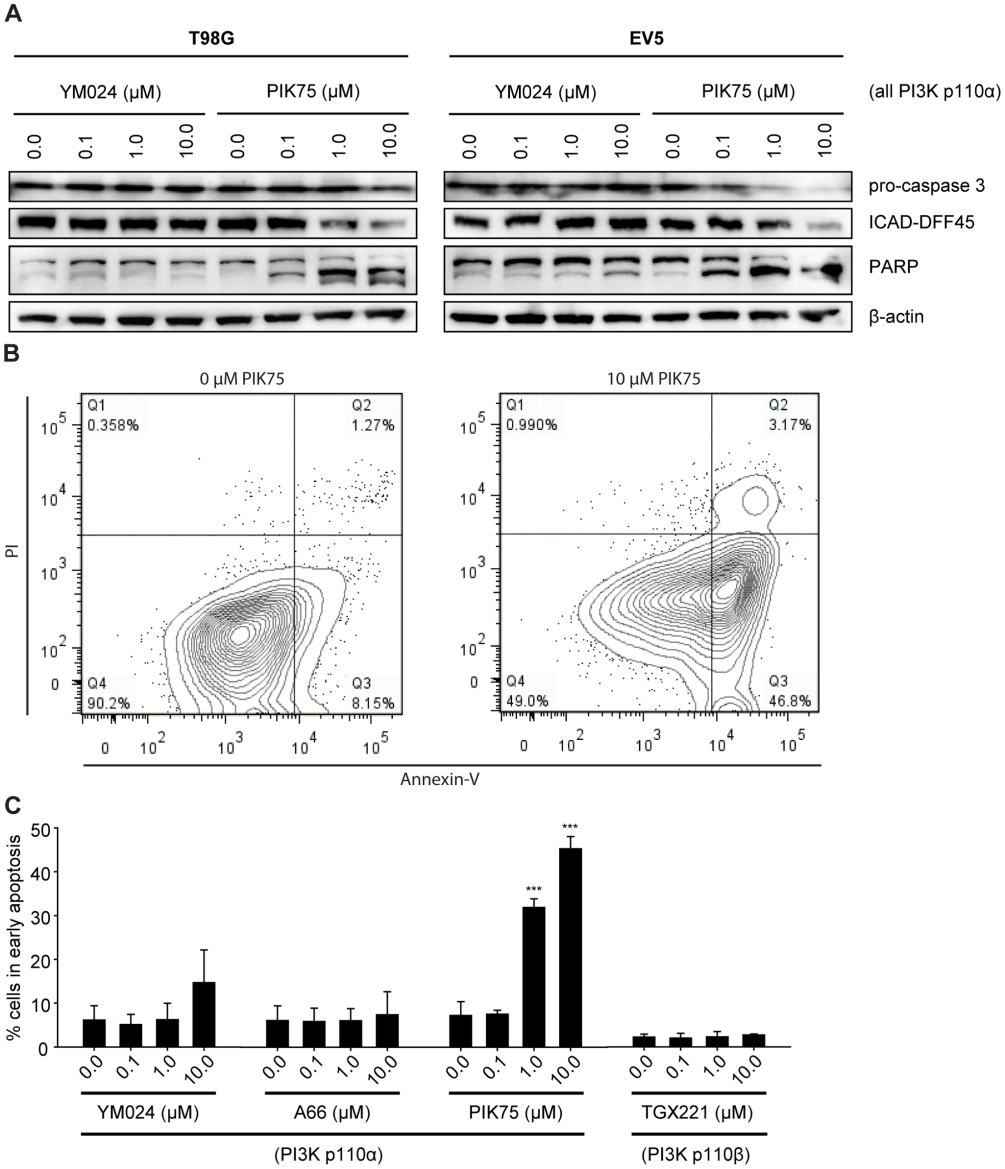
Inhibition of the PI3K isoform p110α with isoform-specific inhibitor PIK75 induces apoptosis. (A) Western blot analysis of apoptosis markers of GBM cells following treatment with increasing concentrations of PI3K p110α-specific inhibitors YM024 or PIK75 (6 h). (B) Flow cytometry analysis of T98G cells after treatment with PI3K p110α-specific inhibitor PIK75 (8 h) stained with Annexin-V and PI. (C) Quantification of Annexin-V-positive/PI-negative T98G cells after treatment with PI3K p110α-specific inhibitors YM024, A66, PIK75, or PI3K p110β-specific inhibitor TGX221 (8 h). Bars represent the means of three or two individual experiments ± standard deviation for PI3K p110α- or PI3K p110β-specific inhibitors, respectively; ***: p≤0.001 compared to 0.0 μM inhibitor as determined by two-sided, one-sample Student’s *t*-tests.

To analyze the cause of reduced cell proliferation of GBM cells upon inhibitor treatment, we performed Western blot analysis of apoptosis markers (caspase 3, ICAD, and cleaved PARP; Figure 3A). According to these blots, the PI3K p110α-specific inhibitor PIK75 induced PARP cleavage and decreased pro-caspase 3 and ICAD-DIFF45 protein levels after 6 h of treatment, indicating thereby that the reduction in cell proliferation is in part due to the induction of apoptosis. The PI3K p110α-specific inhibitor YM024 also induced PARP cleavage at a concentration of 10 μM in one of the GBM cell lines tested. Flow cytometry experiments confirmed the induction of apoptosis by the PI3K p110α-specific inhibitor PIK75, as observed by an increase in Annexin-V-positive/PI-negative cells after 8 h of treatment (Figure 3B and C). Furthermore, the PI3K p110α-specific inhibitor YM024 led to an increase in the number of cells in early apoptosis, but to a lesser extent than PIK75. On the contrary, treatment with the PI3K p110β-specific inhibitor TGX221 did not lead to a significant increase in the number of cells in early apoptosis (Figure 3C).

### PI3K Isoforms p110α and p110β Play a Role in GBM Cell Migration

**Figure 4:**
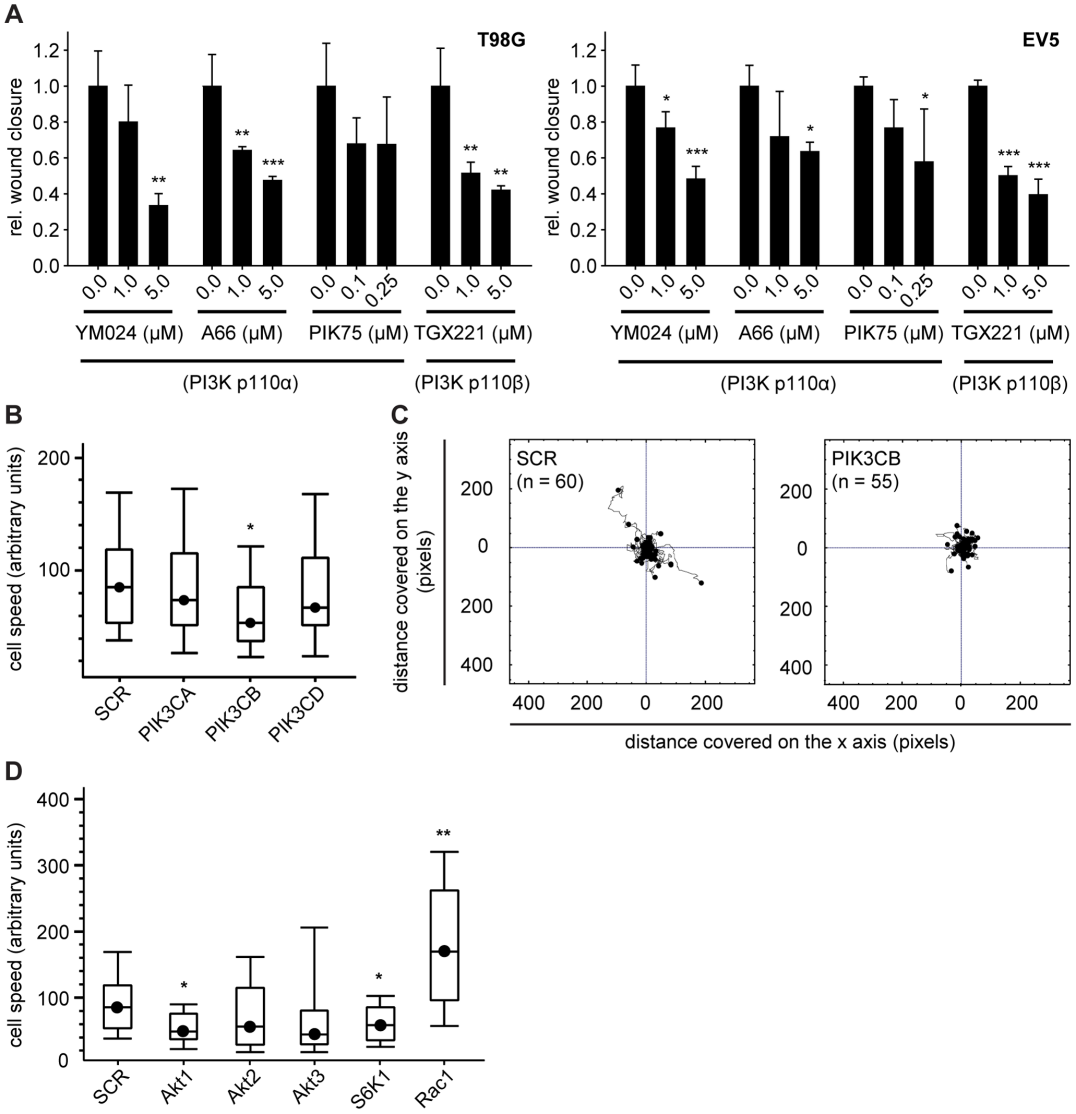
Class I_A_ PI3K isoforms p110α and p110β play a role in GBM cell migration. (A) Analysis of the migratory potential of GBM cells by means of wound healing assays in the presence of PI3K p110α or PI3K p110β-specific inhibitors (18 h). Bars represent the means of three individual experiments ± standard deviation; *: p≤0.05, **: p≤0.01, ***: p≤0.001 compared to 0.0 μM inhibitor as determined by two-sided, one-sample Student’s *t*-tests. (B) Migration speed of T98G cells in a random migration experiment following downregulation of class I_A_ PI3K isoforms by siRNA. *: p≤0.05 compared to SCR non-targeting siRNA as determined by two-sided, one-sample Student’s *t*-test. (C) Distance of migration in a random migration experiment of T98G cells transfected with non-targeting control (SCR) or PI3K p110β-targeting siRNA (PIK3CB). (D) Migration speed of T98G cells in a random migration experiment following downregulation of PI3K/Akt signaling pathway molecules by siRNA. *: p≤0.05, **: p≤0.01 compared to SCR non-targeting siRNA as determined by two-sided, two-sample Student’s *t*-tests.

In order to investigate the role of the different class I_A_ PI3K isoforms in GBM cell migration, we performed *in vitro* wound healing assays by introducing a scratch into a confluent layer of cells. Pharmacological inhibition of PI3K p110α significantly impaired the ability of T98G and EV5 cells to migrate into the open wound area (Figure 4A). Inhibition of PI3K p110β showed even more prominent effects on GBM cell migration, inhibiting wound closure already at lower concentrations (Figure 4A). In contrast, pharmacological inhibition of PI3K p110δ had no significant effect on wound closure (Figure S4B). These results indicate that PI3K isoforms p110α and p110β are involved in the migratory potential of GBM cells.

To complement these studies, we transiently downregulated individual class I_A_ PI3K isoforms in T98G cells and investigated random migration of these cells. Downregulation of the PI3K isoform p110β (*PIK3CB*) negatively affected cell speed when compared to control cells (SCR), while siRNA-targeting of PI3K p110α or PI3K p110δ (*PIK3CA* or *PIK3CD*) had no significant effect (Figure 4B). Further, analysis of the distance covered by T98G cells transfected with siRNA against PI3K p110β revealed a highly restricted area of movement when compared to mock-transfected cells (Figure 4C). Thus, PI3K p110β appears to significantly contribute to the motility of GBM cells.

In order to assess the contribution of downstream signaling components of the PI3K pathway to GBM cell migration, T98G cells were transiently transfected with siRNAs targeting the three Akt isoforms (Akt1-3), the ribosomal protein S6 kinase (S6K1), or the small GTP-binding protein Rac1. Silencing Akt1 and S6K1 significantly reduced the migration speed of T98G cells, while silencing Akt2 or Akt3 did not result in comparable effects (Figure 4D). In contrast, silencing Rac1 significantly enhanced the migration speed of T98G cells (Figure 4D). Thus, Akt1 and S6K1 most likely function downstream of class I_A_ PI3K isoforms in the control of cell migration in GBM cells.

### PI3K p110α and PI3K p110β Inhibition Impairs Activation of the Akt/mTOR Signaling Pathway

**Figure 5:**
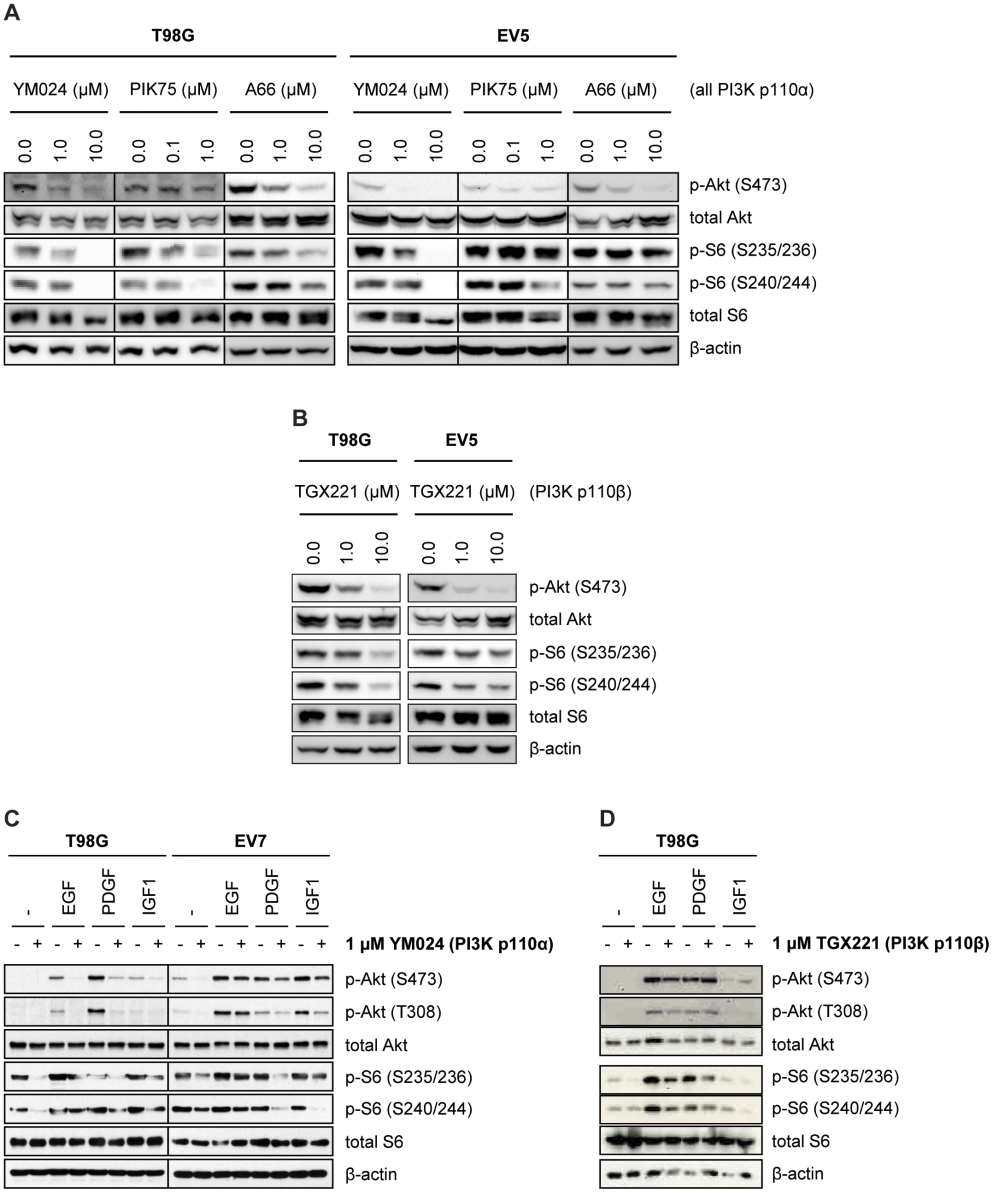
Pharmacological inhibition of PI3K p110α and PI3K p110β impairs downstream signaling. (A) Western blot analysis of basal Akt/mTOR signaling activation by detection of phosphorylated downstream proteins Akt and S6 in GBM cells following treatment with increasing concentrations of PI3K p110α-specific inhibitors YM024, PIK75, or A66 (6 h). (B) Western blot analysis of phosphorylated downstream proteins Akt and S6 protein of GBM cells following treatment with increasing concentrations of the PI3K p110β-specific inhibitor TGX221 (6 h). (C) Growth factor-induced PI3K/Akt signaling activation after pretreatment of T98G and EV7 cells with PI3K p110α-specific inhibitor YM024. (D) Growth factor-induced PI3K/Akt signaling activation after pretreatment of T98G cells with PI3K p110β-specific inhibitor TGX221.

To investigate if pharmacological inhibition of PI3K p110α successfully attenuates basal Akt/mTOR signaling pathway activation, we performed Western blot analysis of GBM cells treated with three different PI3K p110α-specific inhibitors (YM024, A66, or PIK75). Indeed, all three inhibitors led to a concentration-dependent decrease in the phosphorylation levels of downstream Akt and S6 protein (Figure 5A). Additionally, targeting of PI3K p110β with the isoform-specific inhibitor TGX221 also led to a concentration-dependent decrease in the phosphorylation levels of Akt and S6 protein (Figure 5B).

Numerous receptor tyrosine kinases (RTKs) are thought to contribute to the malignant properties of gliomas through autocrine signaling loops. Well documented growth factor systems include the EGF system, the PDGF system and the IGF-I system [9], [42], [43]. We were thus further interested in evaluating the potential of PI3K p110-specific inhibition by YM024 (PI3K p110α) or TGX221 (PI3K p110β) to affect growth factor-induced pathway activation. Indeed, pre-treatment of T98G and EV7 cells with YM024 impaired phosphorylation of Akt and S6 protein in response to EGF, PDGF, or IGF-1 stimulation (Figure 5C). Contrary to pharmacological inhibition of PI3K p110α, treatments with the PI3K p110β-specific inhibitor TGX221 only partially decreased phosphorylation of the ribosomal S6 protein and not to the same extend as the PI3K p110α inhibitor YM024. These results implicate that PI3K p110α plays a crucial role in transmitting signals from activated RTKs and thus could be involved in autocrine/paracrine signaling events in GBM.

Given the known existing cross-talks between the PI3K/Akt/mTOR and the Ras/extracellular signal-regulated kinase (ERK) signaling pathway, we performed Western blot analysis of the GBM cells EV5, T98G, U87 and U251 after treatment with PI3K isoform-specific inhibitors (6 h) and analyzed the phosphorylation levels of ERK. These experiments showed no induction of ERK phosphorylation upon treatment of GBM cells with PI3K isoform-specific inhibitors and thereby indicate no feedback compensation through the Ras/ERK signaling pathway (data not shown).

### Pharmacological Inhibition of PI3K p110α Impairs T98G Tumor Growth in vivo

**Figure 6:**
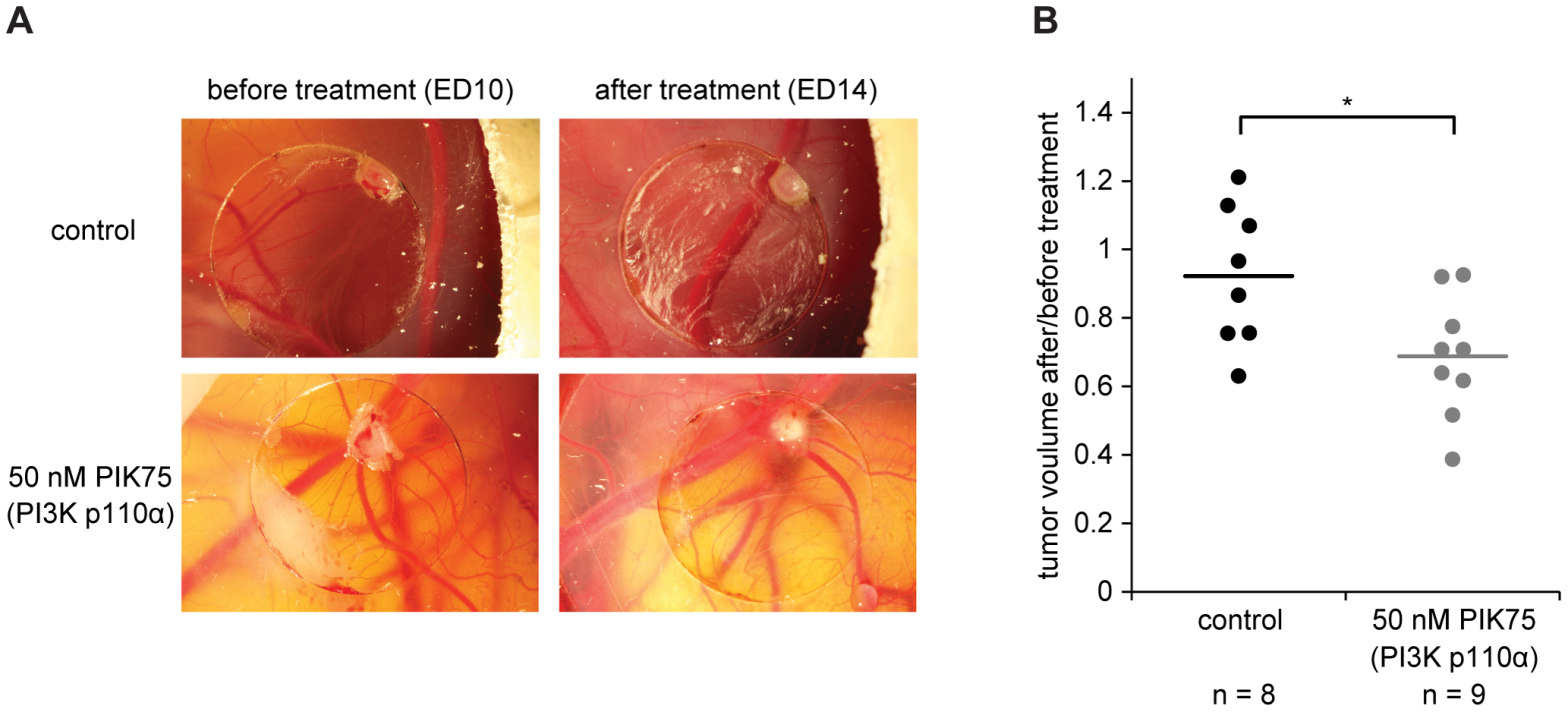
PI3K p110α-specific inhibition of T98G tumors formed on the chick chorioallantoic membrane impairs tumor growth. (A) Representative pictures of T98G tumors formed on the CAM three days post cell application (10 × magnification). Tumors were treated with PIK75 as indicated for four consecutive days. (B) Quantification of changes in tumor volume before and after PIK75 or control treatment. Lines indicate the mean of each group. *: p = 0.02 compared to control treatment as determined by two-sided, two-sample Student’s *t*-test.

To investigate the potential of PI3K p110α-specific inhibition on tumor growth, we performed an *in vivo* CAM assay using T98G GBM cells. Deposition of these cells on the CAM resulted in the formation of solid tumors within three days (Figure 6A, before treatment). Since the PI3K p110α-specific inhibitor PIK75 showed the most potent effects in the *in vitro* experiments, we used this inhibitor in the *in vivo* setting. Indeed, treating the T98G tumors formed on the CAM for four consecutive days with PIK75 led to decreased tumor sizes when compared to control treatment (Figure 6A and B).

## Discussion

The development of novel targeted therapies for GBM is of crucial importance in view of the dismal prognosis of patients with current treatment protocols. It has been recognized that the PI3K/Akt/mTOR pathway is a very promising target for the development of novel anti-cancer therapies in human cancer, including GBM [44], [45]. The most prominent mutations targeting the pathway in GBM are in *PTEN*, *PIK3CA,* and *EGFR* [11]–[13], [18]. To date, the only agents that have been evaluated in GBM clinical trials are allosteric mTOR inhibitors (analogs of rapamycin, rapalogs), which have not shown significant activity, either as single agents or in combination with standard chemotherapy [11], [44]. This may be explained by various factors, including the multiple molecular mechanisms that exist which enable tumors to develop resistance to allosteric mTOR inhibitors [46]. Therefore, agents targeting Akt, PI3K, and mTOR may represent more promising drugs to inhibit this pathway in GBM patients [46], [47]. Indeed, there exist several reports on the pre-clinical activity of Akt, PI3K, and mTOR inhibitors in GBM models, and several clinical trials have been initiated with these novel agents [11], [44], [45]. Concerning PI3K targeting, it has been generally accepted that the dual targeting of PI3K p110α and mTOR is the most potent strategy to inhibit the pathway in a variety of human cancers, including GBM, by circumventing some of the feedback loops which control tumor resistance to mTOR inhibitors [48]. In addition, targeting class I PI3K isoforms has been recently shown to have activity in GBM models, which was dependent on *TP53* status [49]. Targeting the class I_A_ PI3K isoform p110δ was also recently shown to impair the migration of GBM cells [50]. Another set of reports has demonstrated that PI3K p110β may be the most relevant PI3K isoform to target in cancers which have mutations in *PTEN*, such as GBM and prostate cancer [51], [52]. So far there is no consensus on the most relevant predictive biomarkers for PI3K inhibitors in GBM, although there are reports demonstrating that cancers with *PIK3CA* mutations are most sensitive to these agents [53], [54].

In this report, we show by IHC staining of a glioma TMA that PI3K p110α is the class I_A_ PI3K isoform which displays the most consistent expression in GBM patient samples, in contrast to PI3K p110β. Due to the lack of suitable antibodies, it was not possible to assess the expression of PI3K p110δ, which was previously reported to be overexpressed at the mRNA level in primary GBM [13], [22]. In cell lines and *ex vivo* cultures, all three class I_A_ PI3K isoforms were expressed, indicating that GBM cell lines and *ex vivo* cultures may up-regulate the expression of some PI3K isoforms, such as PI3K p110β. When evaluating the contribution of the expression of components of the EGFR/PI3K/Akt/mTOR pathway to patient survival, it was found that phosphorylation of ribosomal S6 protein was the best predictor of reduced patient survival. In addition, co-expression of the EGFR/PI3K p110α/p-S6 module was related to patient survival, indicating that the activation of the pathway can influence GBM outcome. Interestingly, this phenomenon was independent of PTEN and p-Akt status, indicating that there exists a group of GBM tumors in which the EGFR/PI3K/mTOR pathway is active by increased expression of some components, such as EGFR and PI3K p110α. On the other hand, GBM characterized by reduced or absent PTEN expression may rely on another PI3K isoform, such as PI3K p110β [51], [52]. There are several reports which have investigated the prognostic value of mutations in genes involved in the PI3K signaling pathway as well as the activation status of Akt and the ribosomal S6 protein in GBM. While there is no consensus on the prognostic value of *PTEN* mutations or expression, the phosphorylation status of the ribosomal S6 protein has been previously inversely correlated to patient survival [7], [55]–[57].

In view of the consistent expression of PI3K p110α in primary GBM samples and its association with EGFR and mTOR pathway activation, we next evaluated a panel of pharmacological isoform-specific inhibitors in GBM cell lines and *ex vivo* cultures expressing the EGFR/PI3K p110α/p-S6 module. In comparison to the dual PI3K p110α/mTOR inhibitor BEZ235, PI3K p110α-specific inhibitors displayed less potent activity when evaluated on cells growing under adherent and optimal serum conditions. The activity of the three PI3K p110α inhibitors in cells reflected their potency for inhibition of PI3K p110α kinase activity *in vitro*. In addition, some of the *PIK3CA* mutant GBM cell lines SK-MG-26 (H1047Y) responded better to the PI3K p110α-specific inhibitors than GBM cell lines where *PIK3CA* is wild type. *PIK3CA* mutations at position H1047 have been reported to predict sensitivity to inhibitors of the PI3K pathway [53], which may explain the higher sensitivity of the SK-MG-26 (H1047Y) cell line towards PI3K p110α-specific inhibitors. However, the activity of the PI3K p110α inhibitors was not influenced by PTEN status. This was confirmed by the observation that RNAi targeting PI3K p110α partially reduced cell proliferation under adherent and optimal serum conditions. In contrast, when assayed under anchorage-independent conditions, the PI3K p110α and PI3K p110β inhibitors had single agent activity in different GBM cell lines and *ex vivo* cultures. In contrast, PI3K p110δ inhibitors or siRNA did not show a similar broad response in GBM, either under adherent or anchorage-independent growth conditions. Furthermore, the observation that PI3K signaling pathway inhibition impairs anchorage-independent growth of GBM cells, by targeting PI3K p110α or PI3K p110β, complements previously published data investigating the role of Akt3 with respect to anchorage-independent growth in GBM cells [58], [59]. These investigations show that GBM cell lines are dependent on Akt3 by means of siRNA or shRNA downregulation of Akt3 and analysis of anchorage-independent growth in soft agar.

When investigating the role of PI3K isoforms in GBM cell migration, PI3K p110β appeared to play a major role. In contrast to a recent report [50], PI3K p110δ targeting by siRNA or pharmacological inhibitors did not significantly impair GBM cell migration. Contrary to previously published data stating that cell migration of T98G GBM cells is dependent on Akt3 rather than Akt1 or Akt2 [58], our assay showed that T98G GBM cell migration depends on Akt1 rather than Akt2 or Akt3. The discrepancy to this publication could be explained by the different experimental setups: while we were using a random migration assay on plastic with equivalent distribution of nutrients, Endersby *et al.* used a nutrient-driven migration assay through matrigel. Different nutrient environment as well as the presence of matrigel might lead to the expression of different protein levels in the Akt isoforms and might thus result in different cellular responses.

The induction of apoptosis was observed in GBM at high concentrations of the PI3K p110α inhibitors, indicating that single agent targeting of this isoform is not sufficient to induce cell death under adherent conditions. This observation is supported by previous studies, which have demonstrated that the induction of cell cycle arrest is the most prominent mechanism of action of these drugs, when used as single agents under adherent conditions [35]. When evaluated in an *in vivo* model, the PI3K p110α inhibitor PIK75 induced tumor regression of GBM, supporting the view that single agent targeting of PI3K p110α may have anti-tumor effects.

Together our data demonstrate that the PI3K p110α and PI3K p110β isoforms appear to differentially contribute to critical cell responses in GBM, which is important when considering the future use of isoform-specific PI3K inhibitors. Drugs targeting PI3K p110α may be considered for further evaluation in GBM, in particular in tumors which express a functional EGFR/PI3K p110α/p-S6 signaling module. These findings are important, since at present it is unclear which drugs targeting the PI3K/Akt/mTOR pathway have the most favorable profile in glioblastoma patients and there are no robust predictive biomarkers for these agents.

## Supporting Information

Figure S1 **Cell proliferation of GBM cells after targeting of class I_A_ PI3K isoforms.** (A) Cell proliferation of GBM cells in the presence of class I_A_ PI3K isoform-specific inhibitors (72 h). (B) Cell proliferation of GBM cells transiently transfected with siRNA targeting class I_A_ PI3K isoforms p110α (PIK3CA), p110β (PIK3CB), or p110δ (PIK3CD). TOX and SCR siRNAs were used as positive and negative, non-targeting controls, respectively. Curves and bars represent the means of three individual experiments ± standard deviation; *: p≤0.05, **: p≤0.01 compared to SCR non-targeting control siRNA as determined by two-sided, one-sample Student’s *t*-tests.
(TIF)

Figure S2 **Transient transfection of GBM cells with siRNA targeting specific class I_A_ PI3K isoforms.** Transient transfection of T98G (A) and EV5 (B) cells with siRNA targeting class I_A_ PI3K isoforms p110α (PIK3CA), p110β (PIK3CB), or p110δ (PIK3CD) leads to target downregulation at the mRNA level (upper panels) and protein level (lower panels) 48 h post transfection. Bars represent the means of three individual experiments ± standard deviation; **: p≤0.01, ***: p≤0.001 compared to SCR non-targeting control siRNA as determined by two-sided, one-sample Student’s *t*-tests
(TIF)

Figure S3 **PI3K p110α-specific inhibitor treatment of** ***PIK3CA*** **wild type and** ***PIK3CA*** **mutated GBM cell lines.** *PIK3CA* wild type GBM cell line T98G and *PIK3CA* mutated GBM cell lines SK-MG-17 (V344G) and SK-MG-26 (H1047Y) were treated with PI3K p110α-specific inhibitors YM024, PIK75, or A66 (72 h). Curves represent the means of three independent experiments ± standard deviation. *: p≤0.05, **: p≤0.01 compared to T98G cells as determined by two-sided, one-sample Student’s *t*-tests.
(TIF)

Figure S4 **Soft agar colony formation assay and wound healing assay of GBM cells.** (A) Anchorage-independent growth (colony formation in soft agar of GBM cells in the presence of PI3K p110δ-specific inhibitor IC87114 (28 d). (B) Analysis of the migratory potential of GBM cells by means of wound healing assays in the presence of PI3K p110δ-specific inhibitor IC87114 (18 h). Bars represent the means of three individual experiments ± standard deviation.
(TIF)

Table S1 **Demographics and disease characteristics of 74 patients with complete disease and IHC information.**
(PDF)

Table S2 **Univariable analyses of 74 patients with complete disease and IHC information.**
(DOC)

Table S3 **Genetic background information about the GBM cell lines used in this study.**
(PDF)

Table S4 **Additional information about the GBM** ***ex vivo*** **cultures used in this study.**
(PDF)

## References

[1] Central Brain Tumor Registry of the United States CBTRUS statistical report: primary brain and central nervous system tumors diagnosed in the United States in 2004–22007.

[2] Wen PY, Kesari S (2008) Malignant gliomas in adults. N Engl J Med 359: 492–507. 18669428 10.1056/NEJMra0708126

[3] DeAngelis LM (2001) Brain tumors. N Engl J Med 344: 114–123. 11150363 10.1056/NEJM200101113440207

[4] Lee JC, Vivanco I, Beroukhim R, Huang JH, Feng WL, et al (2006) Epidermal growth factor receptor activation in glioblastoma through novel missense mutations in the extracellular domain. PLoS Med 3: e485. 17177598 10.1371/journal.pmed.0030485 PMC1702556

[5] Halatsch ME, Schmidt U, Behnke-Mursch J, Unterberg A, Wirtz CR (2006) Epidermal growth factor receptor inhibition for the treatment of glioblastoma multiforme and other malignant brain tumours. Cancer Treat Rev 32: 74–89. 16488082 10.1016/j.ctrv.2006.01.003

[6] Choe G, Horvath S, Cloughesy TF, Crosby K, Seligson D, et al (2003) Analysis of the phosphatidylinositol 3′-kinase signaling pathway in glioblastoma patients in vivo. Cancer Res 63: 2742–2746. 12782577

[7] Chakravarti A, Zhai G, Suzuki Y, Sarkesh S, Black PM, et al (2004) The prognostic significance of phosphatidylinositol 3-kinase pathway activation in human gliomas. J Clin Oncol 22: 1926–1933. 15143086 10.1200/JCO.2004.07.193

[8] Bowers DC, Fan S, Walter KA, Abounader R, Williams JA, et al (2000) Scatter factor/hepatocyte growth factor protects against cytotoxic death in human glioblastoma via phosphatidylinositol 3-kinase- and AKT-dependent pathways. Cancer Res 60: 4277–4283. 10945642

[9] Chakravarti A, Loeffler JS, Dyson NJ (2002) Insulin-like growth factor receptor I mediates resistance to anti-epidermal growth factor receptor therapy in primary human glioblastoma cells through continued activation of phosphoinositide 3-kinase signaling. Cancer Res 62: 200–207. 11782378

[10] Katso R, Okkenhaug K, Ahmadi K, White S, Timms J, et al (2001) Cellular function of phosphoinositide 3-kinases: implications for development, homeostasis, and cancer. Annu Rev Cell Dev Biol 17: 615–675. 11687500 10.1146/annurev.cellbio.17.1.615

[11] Höland K, Salm F, Arcaro A (2011) The Phosphoinositide 3-Kinase Signaling Pathway as a Therapeutic Target in Grade IV Brain Tumors. Curr Cancer Drug Targets 11: 894–918. 21861842 10.2174/156800911797264743

[12] The Cancer Genome Atlas Research Network (2008) Comprehensive genomic characterization defines human glioblastoma genes and core pathways. Nature 455: 1061–1068. 18772890 10.1038/nature07385 PMC2671642

[13] Knobbe CB, Reifenberger G (2003) Genetic alterations and aberrant expression of genes related to the phosphatidyl-inositol-3′-kinase/protein kinase B (Akt) signal transduction pathway in glioblastomas. Brain Pathol 13: 507–518. 14655756 10.1111/j.1750-3639.2003.tb00481.x PMC8095764

[14] Broderick DK, Di C, Parrett TJ, Samuels YR, Cummins JM, et al (2004) Mutations of PIK3CA in anaplastic oligodendrogliomas, high-grade astrocytomas, and medulloblastomas. Cancer Res 64: 5048–5050. 15289301 10.1158/0008-5472.CAN-04-1170

[15] Hartmann C, Bartels G, Gehlhaar C, Holtkamp N, von Deimling A (2005) PIK3CA mutations in glioblastoma multiforme. Acta Neuropathol 109: 639–642. 15924253 10.1007/s00401-005-1000-1

[16] Stambolic V, Suzuki A, de la Pompa JL, Brothers GM, Mirtsos C, et al (1998) Negative regulation of PKB/Akt-dependent cell survival by the tumor suppressor PTEN. Cell 95: 29–39. 9778245 10.1016/s0092-8674(00)81780-8

[17] Maehama T, Dixon JE (1998) The tumor suppressor, PTEN/MMAC1, dephosphorylates the lipid second messenger, phosphatidylinositol 3,4,5-trisphosphate. J Biol Chem 273: 13375–13378. 9593664 10.1074/jbc.273.22.13375

[18] Samuels Y, Wang Z, Bardelli A, Silliman N, Ptak J, et al (2004) High frequency of mutations of the PIK3CA gene in human cancers. Science 304: 554. 15016963 10.1126/science.1096502

[19] Knobbe CB, Trampe-Kieslich A, Reifenberger G (2005) Genetic alteration and expression of the phosphoinositol-3-kinase/Akt pathway genes PIK3CA and PIKE in human glioblastomas. Neuropathol Appl Neurobiol 31: 486–490. 16150119 10.1111/j.1365-2990.2005.00660.x

[20] Mueller W, Mizoguchi M, Silen E, D’Amore K, Nutt CL, et al (2005) Mutations of the PIK3CA gene are rare in human glioblastoma. Acta Neuropathol 109: 654–655. 15924252 10.1007/s00401-005-1001-0

[21] Gallia GL, Rand V, Siu IM, Eberhart CG, James CD, et al (2006) PIK3CA gene mutations in pediatric and adult glioblastoma multiforme. Mol Cancer Res 4: 709–714. 17050665 10.1158/1541-7786.MCR-06-0172

[22] Mizoguchi M, Nutt CL, Mohapatra G, Louis DN (2004) Genetic alterations of phosphoinositide 3-kinase subunit genes in human glioblastomas. Brain Pathol 14: 372–377. 15605984 10.1111/j.1750-3639.2004.tb00080.x PMC8095817

[23] Hui AB, Lo KW, Yin XL, Poon WS, Ng HK (2001) Detection of multiple gene amplifications in glioblastoma multiforme using array-based comparative genomic hybridization. Lab Invest 81: 717–723. 11351043 10.1038/labinvest.3780280

[24] Louis DN, Ohgaki H, Wiestler OD, Cavenee WK, Burger PC, et al (2007) The 2007 WHO classification of tumours of the central nervous system. Acta Neuropathol 114: 97–109. 17618441 10.1007/s00401-007-0243-4 PMC1929165

[25] Nilsson KP, Ikenberg K, Aslund A, Fransson S, Konradsson P, et al (2010) Structural typing of systemic amyloidoses by luminescent-conjugated polymer spectroscopy. Am J Pathol 176: 563–574. 20035056 10.2353/ajpath.2010.080797 PMC2808065

[26] Van Meir E, Sawamura Y, Diserens AC, Hamou MF, de Tribolet N (1990) Human glioblastoma cells release interleukin 6 in vivo and in vitro. Cancer Res 50: 6683–6688. 2208133

[27] Kaplan EL, Meier P (1958) Nonparametric Estimation from Incomplete Observations. Journal of the American Statistical Association 53: 457–481.

[28] Rodak R, Kubota H, Ishihara H, Eugster HP, Konu D, et al (2005) Induction of reactive oxygen intermediates-dependent programmed cell death in human malignant ex vivo glioma cells and inhibition of the vascular endothelial growth factor production by taurolidine. J Neurosurg 102: 1055–1068. 16028765 10.3171/jns.2005.102.6.1055

[29] Arcaro A, Doepfner KT, Boller D, Guerreiro AS, Shalaby T, et al (2007) Novel role for insulin as an autocrine growth factor for malignant brain tumour cells. Biochem J 406: 57–66. 17506723 10.1042/BJ20070309 PMC1948991

[30] Condliffe AM, Davidson K, Anderson KE, Ellson CD, Crabbe T, et al (2005) Sequential activation of class IB and class IA PI3K is important for the primed respiratory burst of human but not murine neutrophils. Blood 106: 1432–1440. 15878979 10.1182/blood-2005-03-0944

[31] Chaussade C, Rewcastle GW, Kendall JD, Denny WA, Cho K, et al (2007) Evidence for functional redundancy of class IA PI3K isoforms in insulin signalling. Biochem J 404: 449–458. 17362206 10.1042/BJ20070003 PMC1896275

[32] Jackson SP, Schoenwaelder SM, Goncalves I, Nesbitt WS, Yap CL, et al (2005) PI 3-kinase p110beta: a new target for antithrombotic therapy. Nat Med 11: 507–514. 15834429 10.1038/nm1232

[33] Sadhu C, Masinovsky B, Dick K, Sowell CG, Staunton DE (2003) Essential role of phosphoinositide 3-kinase delta in neutrophil directional movement. J Immunol 170: 2647–2654. 12594293 10.4049/jimmunol.170.5.2647

[34] Jamieson S, Flanagan JU, Kolekar S, Buchanan C, Kendall JD, et al (2011) A drug targeting only p110alpha can block phosphoinositide 3-kinase signalling and tumour growth in certain cell types. Biochem J 438: 53–62. 21668414 10.1042/BJ20110502 PMC3174055

[35] Maira SM, Stauffer F, Brueggen J, Furet P, Schnell C, et al (2008) Identification and characterization of NVP-BEZ235, a new orally available dual phosphatidylinositol 3-kinase/mammalian target of rapamycin inhibitor with potent in vivo antitumor activity. Mol Cancer Ther 7: 1851–1863. 18606717 10.1158/1535-7163.MCT-08-0017

[36] Belkaid A, Copland IB, Massillon D, Annabi B (2006) Silencing of the human microsomal glucose-6-phosphate translocase induces glioma cell death: potential new anticancer target for curcumin. FEBS Lett 580: 3746–3752. 16777101 10.1016/j.febslet.2006.05.071

[37] Gebäck T, Schulz MM, Koumoutsakos P, Detmar M (2009) TScratch: a novel and simple software tool for automated analysis of monolayer wound healing assays. Biotechniques 46: 265–274. 19450233 10.2144/000113083

[38] Katso RM, Pardo OE, Palamidessi A, Franz CM, Marinov M, et al (2006) Phosphoinositide 3-Kinase C2beta regulates cytoskeletal organization and cell migration via Rac-dependent mechanisms. Mol Biol Cell 17: 3729–3744. 16775008 10.1091/mbc.E05-11-1083 PMC1593155

[39] Hagedorn M, Javerzat S, Gilges D, Meyre A, de Lafarge B, et al (2005) Accessing key steps of human tumor progression in vivo by using an avian embryo model. Proc Natl Acad Sci U S A 102: 1643–1648. 15665100 10.1073/pnas.0408622102 PMC547849

[40] Weigelt B, Downward J (2012) Genomic Determinants of PI3K Pathway Inhibitor Response in Cancer. Front Oncol 2: 109. 22970424 10.3389/fonc.2012.00109 PMC3431500

[41] Gymnopoulos M, Elsliger MA, Vogt PK (2007) Rare cancer-specific mutations in PIK3CA show gain of function. Proc Natl Acad Sci U S A 104: 5569–5574. 17376864 10.1073/pnas.0701005104 PMC1838453

[42] Nicholas MK, Lukas RV, Jafri NF, Faoro L, Salgia R (2006) Epidermal growth factor receptor - mediated signal transduction in the development and therapy of gliomas. Clin Cancer Res 12: 7261–7270. 17189397 10.1158/1078-0432.CCR-06-0874

[43] Puputti M, Tynninen O, Sihto H, Blom T, Maenpaa H, et al (2006) Amplification of KIT, PDGFRA, VEGFR2, and EGFR in gliomas. Mol Cancer Res 4: 927–934. 17189383 10.1158/1541-7786.MCR-06-0085

[44] Lino MM, Merlo A (2011) PI3Kinase signaling in glioblastoma. J Neurooncol 103: 417–427. 21063898 10.1007/s11060-010-0442-z PMC3116122

[45] Zhang S, Yu D (2010) PI(3)king apart PTEN’s role in cancer. Clin Cancer Res 16: 4325–4330. 20622047 10.1158/1078-0432.CCR-09-2990

[46] Carew JS, Kelly KR, Nawrocki ST (2011) Mechanisms of mTOR inhibitor resistance in cancer therapy. Target Oncol 6: 17–27. 21547705 10.1007/s11523-011-0167-8

[47] Akhavan D, Cloughesy TF, Mischel PS (2010) mTOR signaling in glioblastoma: lessons learned from bench to bedside. Neuro Oncol 12: 882–889. 20472883 10.1093/neuonc/noq052 PMC2940679

[48] Fan QW, Knight ZA, Goldenberg DD, Yu W, Mostov KE, et al (2006) A dual PI3 kinase/mTOR inhibitor reveals emergent efficacy in glioma. Cancer Cell 9: 341–349. 16697955 10.1016/j.ccr.2006.03.029 PMC2925230

[49] Koul D, Fu J, Shen R, LaFortune TA, Wang S, et al (2012) Antitumor activity of NVP-BKM120–a selective pan class I PI3 kinase inhibitor showed differential forms of cell death based on p53 status of glioma cells. Clin Cancer Res 18: 184–195. 22065080 10.1158/1078-0432.CCR-11-1558 PMC3785365

[50] Luk SK, Piekorz RP, Nurnberg B, Tony To SS (2012) The catalytic phosphoinositol 3-kinase isoform p110delta is required for glioma cell migration and invasion. Eur J Cancer 48: 149–157. 22079609 10.1016/j.ejca.2011.09.006

[51] Jia S, Liu Z, Zhang S, Liu P, Zhang L, et al (2008) Essential roles of PI(3)K-p110beta in cell growth, metabolism and tumorigenesis. Nature 454: 776–779. 18594509 10.1038/nature07091 PMC2750091

[52] Wee S, Wiederschain D, Maira SM, Loo A, Miller C, et al (2008) PTEN-deficient cancers depend on PIK3CB. Proc Natl Acad Sci U S A 105: 13057–13062. 18755892 10.1073/pnas.0802655105 PMC2529105

[53] Janku F, Wheler JJ, Naing A, Falchook GS, Hong DS, et al.. (2012) PIK3CA mutation H1047R is associated with response to PI3K/AKT/mTOR signaling pathway inhibitors in early phase clinical trials. Cancer Res. 10.1158/0008-5472.CAN-12-1726 PMC3537862 23066039

[54] Maira SM, Pecchi S, Huang A, Burger M, Knapp M, et al (2012) Identification and Characterization of NVP-BKM120, an Orally Available Pan-Class I PI3-Kinase Inhibitor. Mol Cancer Ther 11: 317–328. 22188813 10.1158/1535-7163.MCT-11-0474

[55] Kim B, Myung JK, Seo JH, Park CK, Paek SH, et al (2010) The clinicopathologic values of the molecules associated with the main pathogenesis of the glioblastoma. J Neurol Sci 294: 112–118. 20441994 10.1016/j.jns.2010.03.019

[56] McBride SM, Perez DA, Polley MY, Vandenberg SR, Smith JS, et al (2010) Activation of PI3K/mTOR pathway occurs in most adult low-grade gliomas and predicts patient survival. J Neurooncol 97: 33–40. 19705067 10.1007/s11060-009-0004-4 PMC2814032

[57] Ermoian RP, Furniss CS, Lamborn KR, Basila D, Berger MS, et al (2002) Dysregulation of PTEN and protein kinase B is associated with glioma histology and patient survival. Clin Cancer Res 8: 1100–1106. 12006525

[58] Endersby R, Zhu X, Hay N, Ellison DW, Baker SJ (2011) Nonredundant functions for Akt isoforms in astrocyte growth and gliomagenesis in an orthotopic transplantation model. Cancer Res 71: 4106–4116. 21507933 10.1158/0008-5472.CAN-10-3597 PMC3118569

[59] Mure H, Matsuzaki K, Kitazato KT, Mizobuchi Y, Kuwayama K, et al (2010) Akt2 and Akt3 play a pivotal role in malignant gliomas. Neuro Oncol 12: 221–232. 20167810 10.1093/neuonc/nop026 PMC2940586

