# Supplementary figures and images for: Targeting Class IA PI3K Isoforms Selectively Impairs Cell Growth, Survival, and Migration in Glioblastoma
Source: PLoS One. 2014 Apr 9;9(4):e94132. doi: 10.1371/journal.pone.0094132 (PMC3981776; doi:10.1371/journal.pone.0094132)

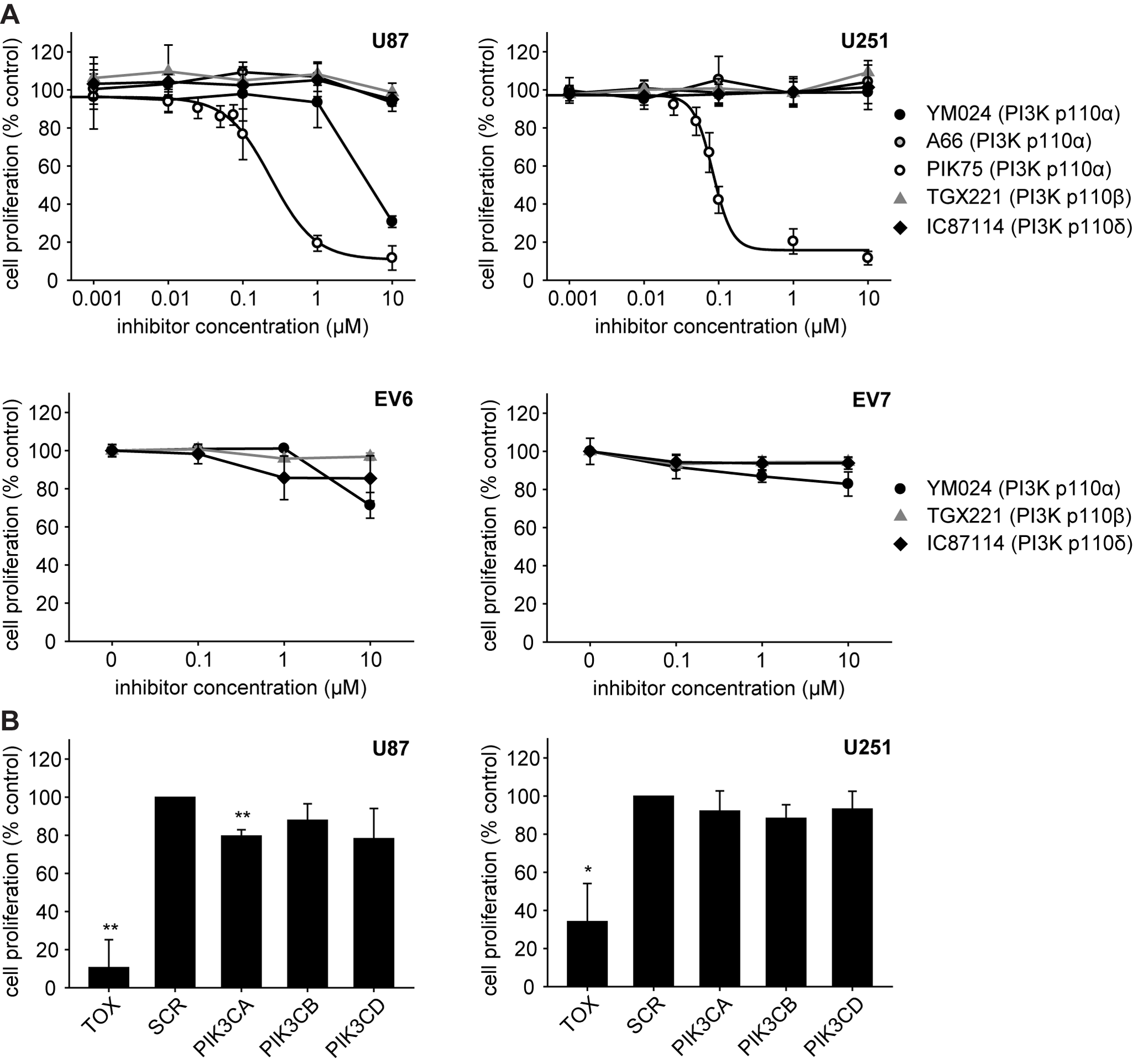

Supplement: Figure S1 — Cell proliferation of GBM cells after targeting of class IA PI3K isoforms. (A) Cell proliferation of GBM cells in the presence of class IA PI3K isoform-specific inhibitors (72 h). (B) Cell proliferation of GBM cells transiently transfected with siRNA targeting class IA PI3K isoforms p110α (PIK3CA), p110β (PIK3CB), or p110δ (PIK3CD). TOX and SCR siRNAs were used as positive and negative, non-targeting controls, respectively. Curves and bars represent the means of three individual experiments ± standard deviation; *: p≤0.05, **: p≤0.01 compared to SCR non-targeting control siRNA as determined by two-sided, one-sample Student’s t-tests. (TIF) [file pone.0094132.s001.tif]

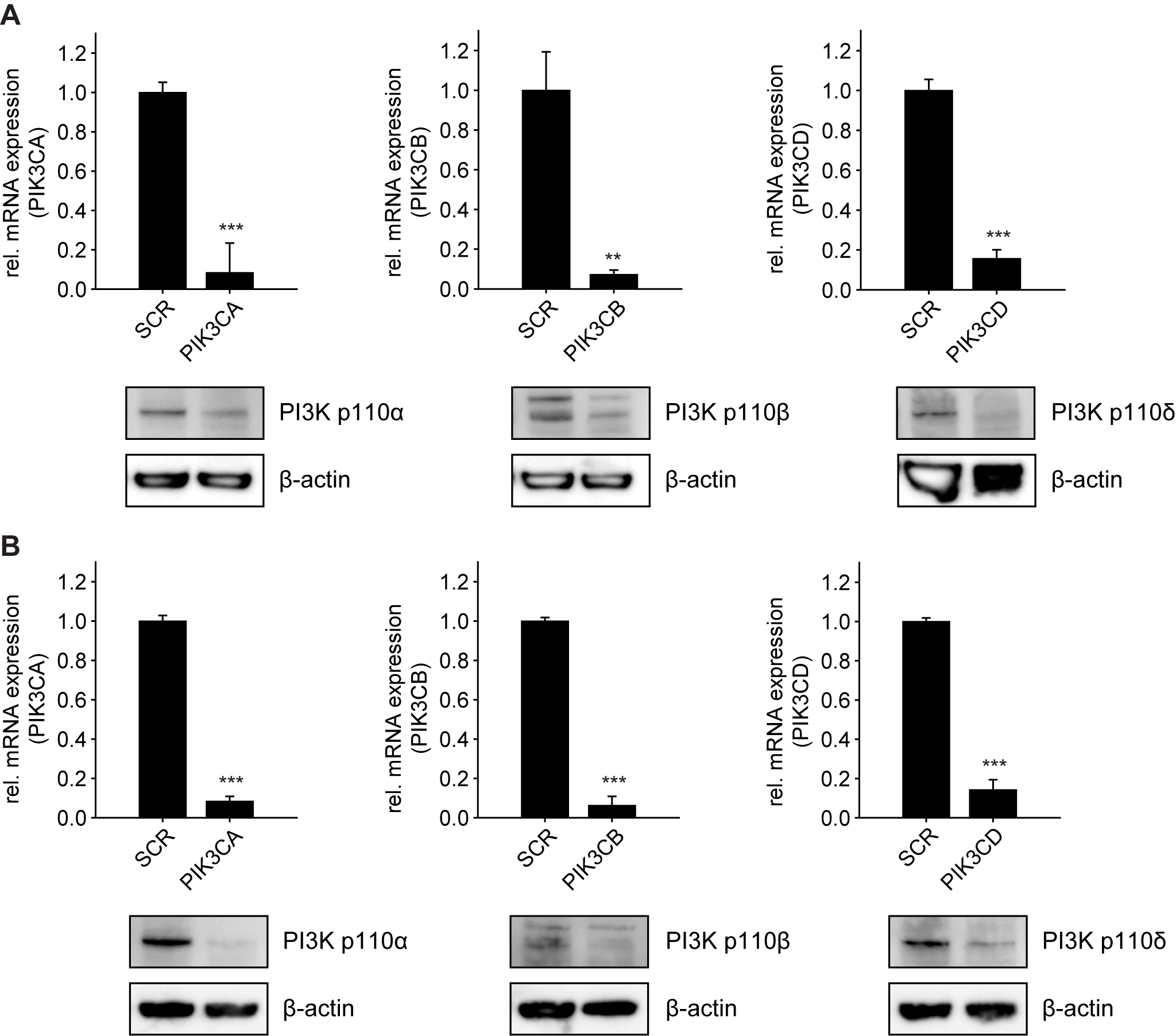

Supplement: Figure S2 — Transient transfection of GBM cells with siRNA targeting specific class IA PI3K isoforms. Transient transfection of T98G (A) and EV5 (B) cells with siRNA targeting class IA PI3K isoforms p110α (PIK3CA), p110β (PIK3CB), or p110δ (PIK3CD) leads to target downregulation at the mRNA level (upper panels) and protein level (lower panels) 48 h post transfection. Bars represent the means of three individual experiments ± standard deviation; **: p≤0.01, ***: p≤0.001 compared to SCR non-targeting control siRNA as determined by two-sided, one-sample Student’s t-tests (TIF) [file pone.0094132.s002.tif]

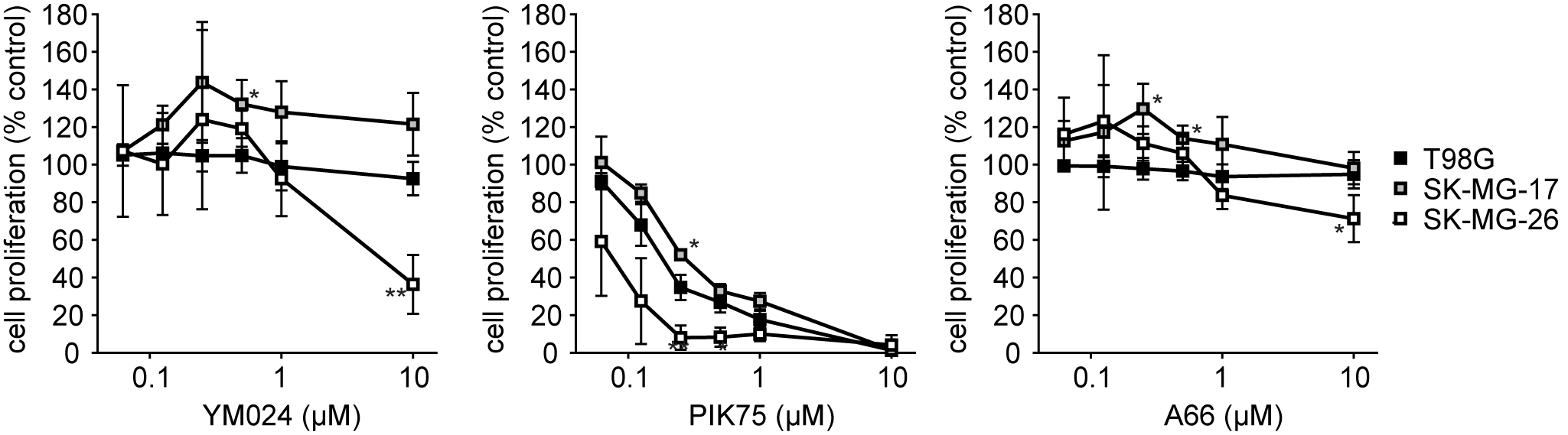

Supplement: Figure S3 — PI3K p110α-specific inhibitor treatment of PIK3CA wild type and PIK3CA mutated GBM cell lines. PIK3CA wild type GBM cell line T98G and PIK3CA mutated GBM cell lines SK-MG-17 (V344G) and SK-MG-26 (H1047Y) were treated with PI3K p110α-specific inhibitors YM024, PIK75, or A66 (72 h). Curves represent the means of three independent experiments ± standard deviation. *: p≤0.05, **: p≤0.01 compared to T98G cells as determined by two-sided, one-sample Student’s t-tests. (TIF) [file pone.0094132.s003.tif]

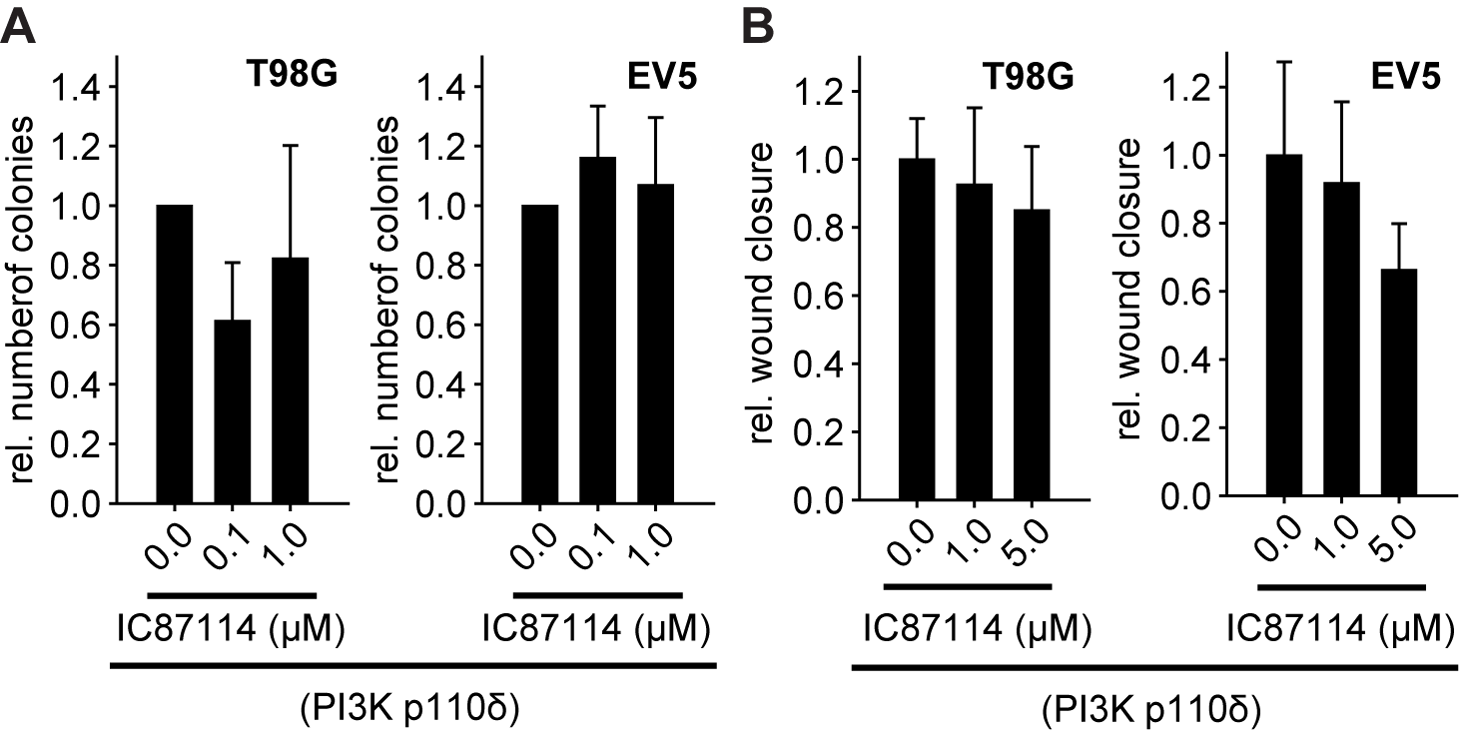

Supplement: Figure S4 — Soft agar colony formation assay and wound healing assay of GBM cells. (A) Anchorage-independent growth (colony formation in soft agar of GBM cells in the presence of PI3K p110δ-specific inhibitor IC87114 (28 d). (B) Analysis of the migratory potential of GBM cells by means of wound healing assays in the presence of PI3K p110δ-specific inhibitor IC87114 (18 h). Bars represent the means of three individual experiments ± standard deviation. (TIF) [file pone.0094132.s004.tif]
