# Supplementary material for: Targeting Class IA PI3K Isoforms Selectively Impairs Cell Growth, Survival, and Migration in Glioblastoma
Source: PLoS One. 2014 Apr 9;9(4):e94132. doi: 10.1371/journal.pone.0094132 (PMC3981776; doi:10.1371/journal.pone.0094132)
Supplement: Table S1 — Demographics and disease characteristics of 74 patients with complete disease and IHC information. (PDF) [file pone.0094132.s005.pdf]

Supplementary Table S1. Demographics and disease characteristics of 74 patients with complete disease and IHC information.

characteristics		number of patients (n = 74)
**gender**	female	26 (35.1%)
	male	48 (64.9%)
**age (years)**	median age at diagnosis (range)	50.0 (1.0 - 80.0)
**WHO grade**	I	7 (9.4%)
	II	8 (10.8%)
	III	17 (23.0%)
	IV	42 (56.8%)
**status**	alive	20 (27.0%)
	dead	54 (73.0)

WHO: world health organization
