# Supplementary material for: Targeting Class IA PI3K Isoforms Selectively Impairs Cell Growth, Survival, and Migration in Glioblastoma
Source: PLoS One. 2014 Apr 9;9(4):e94132. doi: 10.1371/journal.pone.0094132 (PMC3981776; doi:10.1371/journal.pone.0094132)
Supplement: Table S2 — Univariable analyses of 74 patients with complete disease and IHC information. (DOC) [file pone.0094132.s006.doc]

**Supplementary Table S2.** Univariable analyses of 74 patients with complete disease and IHC information.

| **parameter** |  | **number of patients** | **2-year OS rate** | **95% CI** | **p-value of log-rank test** |
| --- | --- | --- | --- | --- | --- |
| **gender** | female | 26 | 38.5% | 19.9 - 57.1% | 0.2259 |
|  | male | 48 | 44.7% | 30.5 - 58.9% |  |
| **WHO grade** | > II | 59 | 32.2% | 20.3 - 44.1% | < 0.001 |
|  | ≤ II | 15 | 85.7% | 67.4 - 100% |  |
| **PI3K p110α** | > 0 | 54 | 41.5% | 28.2 - 54.8% | 0.5436 |
|  | = 0 | 20 | 45.0% | 23.2 - 66.8% |  |
| **p-S6 (S235/236)** | > 0 | 31 | 22.6% | 7.9 - 37.3% | 0.0043 |
|  | = 0 | 43 | 57.1% | 42.2 - 72.0% |  |
| **S6** | > 0 | 9 | 11.1% | 0.0 - 31.7% | 0.0216 |
|  | = 0 | 65 | 46.9% | 34.7 - 59.1% |  |
| **p-Akt (s473)** | > 0 | 1 | 0.0% |  | < 0.001 |
|  | = 0 | 73 | 43.1% | 31.7 - 54.5% |  |
| **Akt** | > 0 | 24 | 41.7% | 22.1 - 61.3% | 0.6720 |
|  | = 0 | 50 | 42.9% | 29.0 - 56.8% |  |
| **EGFR** | > 0 | 59 | 37.9% | 25.4 - 50.4% | 0.0451 |
|  | = 0 | 15 | 60.0% | 35.1 - 84.9% |  |
| **PTEN** | > 0 | 15 | 28.6% | 4.3 - 49.1% | 0.6404 |
|  | = 0 | 57 | 46.4% | 33.4 - 59.5% |  |
| OS: overall survival, CI: confidence interval, WHO: word health organization | | | | | |
