# Supplementary material for: Targeting Class IA PI3K Isoforms Selectively Impairs Cell Growth, Survival, and Migration in Glioblastoma
Source: PLoS One. 2014 Apr 9;9(4):e94132. doi: 10.1371/journal.pone.0094132 (PMC3981776; doi:10.1371/journal.pone.0094132)
Supplement: Table S3 — Genetic background information about the GBM cell lines used in this study. (PDF) [file pone.0094132.s007.pdf]

**Supplementary Table S3.** Genetic background information about the GBM cell lines used in this study.

| **cell line** | ***PTEN*** [a,b] | ***TP53*** [a] | ***PIK3CA*** [c] | ***EGFR* amplification** | **MGMT expression** [d,e,f] |
|---|---|---|---|---|---|
| **LN215** | mut | mut | n.a. | n.a. | n.a. |
| **LN229** | wt | mut | wt | No | no |
| **LN319** | mut | mut | n.a. | n.a. | yes |
| **U87** | mut | wt | wt | no | no |
| **U251** | mut | mut | wt | no | no |
| **T98G** | mut | mut | wt | no | yes |

mut: mutated, wt: wild type, n.a.: not available; (a) Ishii N, Maier D, Merlo A, Tada M, Sawamura Y, et al. (1999) Frequent co-alterations of TP53, p16/CDKN2A, p14ARF, PTEN tumor suppressor genes in human glioma cell lines. Brain Pathol 9: 469-479. (b) Furnari FB, Lin H, Huang HS, Cavenee WK (1997) Growth suppression of glioma cells by PTEN requires a functional phosphatase catalytic domain. Proc Natl Acad Sci U S A 94: 12479-84. (c) Kita D, Yonekawa Y, Weller M, Ohgaki H (2007) PIK3CA alterations in primary (de novo) and secondary glioblastomas. Acta Neuropathol 113: 295-302. (d) Yoshino A, Ogino A, Yachi K, Ohta T, Fukushima T, et al. (2010) Gene expression profiling predicts response to temozolomide in malignant gliomas. Int J Oncol 36: 1367-77. (e) van Nifterik KA, van den Berg J, van der Meide WF, Ameziane N, Wedekind LE, et al. (2010) Absence of the MGMT protein as well as methylation of the MGMT promoter predict the sensitivity for temozolomide. Br J Cancer 103: 29-35. (f) Hermisson M, Klumpp A, Wick W, Wischhusen J, Nagel G, et al. (2006) O6-methylguanine DNA methyltransferase and p53 status predict temozolomide sensitivity in human malignant glioma cells. J Neurochem 96: 766-76.
