# Supplementary material for: Targeting Class IA PI3K Isoforms Selectively Impairs Cell Growth, Survival, and Migration in Glioblastoma
Source: PLoS One. 2014 Apr 9;9(4):e94132. doi: 10.1371/journal.pone.0094132 (PMC3981776; doi:10.1371/journal.pone.0094132)
Supplement: Table S4 — Additional information about the GBM ex vivo cultures used in this study. (PDF) [file pone.0094132.s008.pdf]

Supplementary Table S4. Additional information about the GBM *ex vivo* cultures used in this study.

***ex vivo* culture**	**WHO grade**	**age (years)**	**gender**
**EV1**	III	37	female
**EV2**	IV	77	male
**EV3**	II	42	female
**EV4**	IV	59	male
**EV5**	III	61	male
**EV6**	IV	65	male
**EV7**	IV	54	male

WHO: word health organization
